# Global transmission and evolutionary dynamics of the Chikungunya virus

**DOI:** 10.1017/S0950268820000497

**Published:** 2020-02-19

**Authors:** F. Deeba, M. S. H. Haider, A. Ahmed, A. Tazeen, M. I. Faizan, N. Salam, T. Hussain, S. F. Alamery, S. Parveen

**Affiliations:** 1 Centre for Interdisciplinary Research in Basic Sciences, Jamia Millia Islamia, New Delhi, India; 2 Centre of Excellence in Biotechnology Research, College of Science, King Saud University, Riyadh, Saudi Arabia; 3 Protein Research Chair, Department of Biochemistry, College of Science, King Saud University, Riyadh, Saudi Arabia; 4 Department of Pathology, College of Medicine, Al-Imam Mohammad Ibn Saud Islamic University (IMSIU), Riyadh, Saudi Arabia

**Keywords:** Bayesian analysis, Chikungunya virus, docking, entropy, networking, phylogenetics, selection pressure

## Abstract

Chikungunya virus (CHIKV) is a re-emerging pathogen of global importance. We attempted to gain an insight into the organisation, distribution and mutational load of the virus strains reported from different parts of the world. We describe transmission dynamics and genetic characterisation of CHIKV across the globe during the last 65 years from 1952 to 2017. The evolutionary pattern of CHIKV was analysed using the E1 protein gene through phylogenetic, Bayesian and Network methods with a dataset of 265 sequences from various countries. The time to most recent common ancestor of the virus was estimated to be 491 years ago with an evolutionary rate of 2.78 × 10^−4^ substitutions/site/year. Genetic characterisation of CHIKV strains was carried out in terms of variable sites, selection pressure and epitope mapping. The neutral selection pressure on the E1 gene of the virus suggested a stochastic process of evolution. We identified six potential epitope peptides in the E1 protein showing substantial interaction with human MHC-I and MHC-II alleles. The present study augments global epidemiological and population dynamics of CHIKV warranting undertaking of appropriate control measures. The identification of epitopic peptides can be useful in the development of epitope-based vaccine strategies against this re-emerging viral pathogen.

## Introduction

Chikungunya fever is caused by Chikungunya virus (CHIKV), an arboviral pathogen that has caused numerous outbreaks across the globe. It has been suggested that CHIKV probably originated in the Central/East Africa probably around 300–500 years ago [1, 2]. Phylogenetically, CHIKV consists of three separate clades namely (i) The ‘West African’, (ii) ‘Asian’ and (iii) ‘East Central South African’ (ECSA) [3].

**Fig. 1.**
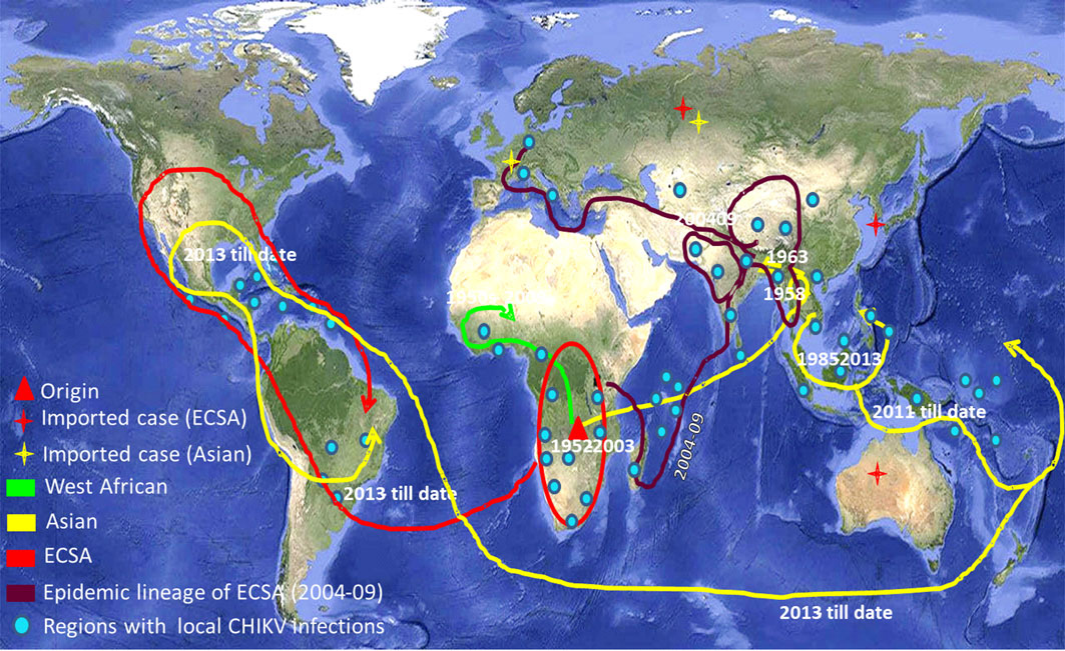
The world map showing the distribution of different lineages of the Chikungunya virus. Regions with the evidence of well-established CHIKV circulation are circled whereas imported cases of CHIKV are denoted by stars in the map. (The map was downloaded from the site: https://commons.wikimedia.org/wiki/Atlas_of_the_world#/media/File:Whole_world_-_land_and_oceans_12000.jpg.)

CHIKV has now been recognised as a global pathogen due to its presence in a wide geographical range [3]. Increased global travel from CHIKV endemic regions has been the major cause of dispersal of the virus to non-endemic regions of the Americas and Europe. CHIKV has succeeded to maintain its mosquito–human life cycle causing outbreaks of autochthonous cases in many geographical regions including the American continents. The virus has caused some autochthonous transmissions in a few parts of the USA during 2014–15 [4]. As the situation stands today, there is no local transmission of CHIKV in the USA currently. The virus has adapted itself to both *Aedes aegypti* and *Aedes albopictus* providing itself an alternate vector and introduction of the disease to previously unexposed populations [5, 6]. The genomic analyses of CHIKV sequences reported from India during the 2009–2010 outbreak revealed mutations in the structural and non-structural regions that contribute to the adaptations of the virus to locally available vector populations [7, 8]. CHIKV is currently circulating in around 100 countries worldwide as described by the Centre for Disease Control and Prevention (CDC) (Fig. 1). The virus has caused many epidemics with the co-circulation of ECSA (East Central and South African) and Asian genotypes, affecting millions of people [9, 10]. The re-emergence of this virus is probably due to mutational changes, increased efficiency of vector transmission, immunologically naive populations, enhanced global dissemination, inadequate public health infrastructure, unforeseen environmental and social factors [11].

Characterisation of the circulating strains is envisaged to be useful for the control and prevention of the infection. We undertook global distribution and evolutionary analysis of CHIKV using phylogenetic, networking and Bayesian methods. This also included mutational analysis of the E1 gene, its variable sites and epitope mapping. We also desired to assess if there is a set pattern of the emergence of Chikungunya fever in different geographical regions. This information can be useful for the prevention and control of CHIKV outbreaks globally. Transmission and evolutionary analysis of the virus will help elucidate host–pathogen dynamics during the course of CHIKV infections in humans.

## Materials and methods

### DNA sequences

The sequences for the present study were taken from different countries at varying time intervals or from the same country at different times in order to avoid the repetition of similar sequences. A total of 265 such sequences (latest available till March 2019) of the partial E1 protein gene of CHIKV were downloaded after an extensive search in GenBank. The dataset also included 153 unique sequences that were used for Bayesian analysis. The details of the sequences used in the study are in Supplementary Table S1.

### Phylogenetic analysis

All the 265 sequences were used for the phylogenetic analysis. Sequences were aligned with using BioEdit (7.2.5) software [12]. Phylogenetic tree was constructed in MEGA X 10.1.5 software with maximum likelihood method with a bootstrap value of 1000 replicates [13]. The S27 strain (GenBank Accession number AF369024) was used as the reference strain of CHIKV.

### Network analysis

The investigation of variation among different sequences, evolutionary pattern and origin of the virus on the basis of divergence of new strains from the parent strain (first isolated strains from 1953 isolates) was also done with all the 265 sequences. These evolutionary relationships were predicted using Network 5.0 software that involves convergent evolution, recombination polymorphism and microevolution in nucleotide sequences [14, 15]. The median joining method was chosen to draw the network. The alignment file was prepared in DnaSP v. 5.10.01 for further use in network analysis [16].

### Bayesian Markov Chain Monte-Carlo (MCMC) analysis

The identification of the time to most recent common ancestor (tMRCA) and rate of substitution provides information about the pattern of evolution and origin of CHIKV. Therefore, the dataset of 153 unique E1 gene sequences was checked for different evolutionary models using the program Model Test 3.7 (coupled with PAUP 4.0b 10) to find the best fit model of substitution for the sequence alignment [17, 18]. GTR (General Time Reversible) as a nucleotide substitution model and G (Gamma distribution) with four rate categories were chosen as the site rate variation model (GTR + G4) for the evolutionary analysis. The tMRCA and nucleotide substitution rate were determined using BEASTv1.8.3 software [19]. The constant size population, exponential growth and Bayesian skyline coalescent tree priors (demographic models) in both strict as well as relaxed molecular clocks were used for the analysis. The MCMC chain was run for 200 million steps with parameter values sampling at every 20 000 steps for all the six different combinations of demographic models and molecular clocks. Log marginal likelihoods were determined by path sampling and stepping stone sampling values [20]. The best combination as suggested by the Bayes factor was analysed in Tracer v1.6 in which the ESS values (effective size sampling) of all the parameters were ensured to be ≥200 in the log file [21]. The Tree Annotator1.8.3 available in BEAST package was used to generate the maximum clade credibility tree and the visualisation of the tree file was done in FigTree v1.4.2 [21]. The Bayesian skyline plot of 40 unique strains from India was also generated using the same BEAST package.

### Shannon entropy analysis

The Shannon entropy analysis was done to identify the highly variable sites within the amino acid sequences with high entropy values. BioEdit (7.2.5) software was used for the Shannon entropy analysis [12, 22]. A cut-off value of 0.2 was set and sites with values >0.2 were considered to be variable [23].

### Selection pressure analysis

The selection pressure analysis of partial E1 gene sequences of all the 153 unique sequences was done using Datamonkey online server (https://www.datamonkey.org/) [24]. Estimation of dN/dS (ratio of non-synonymous to synonymous mutations) was done using HKY85, F81 and REV nucleotide substitution models each under three different approaches that are single likelihood ancestor counting (SLAC), fixed effects likelihood (FEL) and inverse FEL (IFEL) methods [25].

## Epitope mapping

### Identification of B-cell epitopes

The complete E1 sequence of the S27 prototype strain of CHIKV (AF369024) was used for the analysis. The IEDB (Immune Epitope DataBase) was used for the analysis of B-cell antigenicity [26]. Prediction of linear epitopes in the amino acid sequence of E1 protein for B cells was done using six different propensity scale methods available with the IEDB.

### Identification of T-cell epitopes

The T-cell epitope prediction was done through IEDB tools. The MHC-I epitope prediction included the identification of 9mer peptides in the E1 protein that could possibly be the CD8+ T-cell epitopes. Various HLA A, B and C associated epitopes with binding affinity <200 nm (IC50<200) were shortlisted by NetMHCpan tool [27]. These epitopes were further analysed using combined predictor (combined score of proteosomal cleavage and transporter associated with antigen processing (TAP) score), NetCTL (Cytotoxic T Lymphocyte), MHC-NP (Naturally processed Peptides by the MHC) and conservancy and immunogenicity scores. The epitope prediction for MHC class II molecules was done using NetMHCIIpan. All 9mer peptides with IC50<50 were considered to be potential epitopes. These were further analysed using PREDIVAC online tool to reach the most specific epitopes (http://predivac.biosci.uq.edu.au).

### Docking of selected epitopes

The tertiary structures of potential epitopes selected through IEDB epitope analysis were used to dock with interacting alleles having tertiary structure available in the database to study the interaction using AutoDock4.0 software [28]. Here ‘interacting alleles’ are referred to the alleles shortlisted for having maximum interaction scores with the peptides in E1 protein using the results of IEDB server analysis. Docking was done using the methodology previously described [29]

## Results

### Phylogenetic analysis

**Fig. 2.**
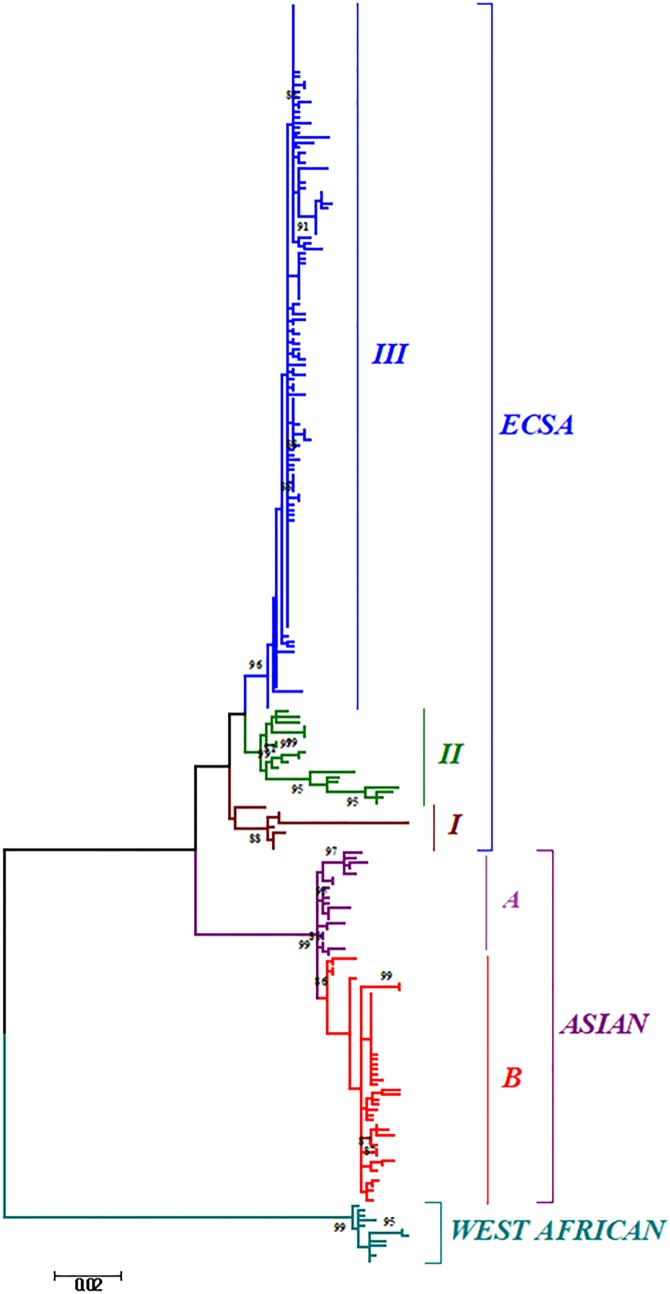
The ML phylogenetic tree of the Chikungunya virus. The tree was generated by 1000 bootstrap values using partial E1 gene sequences. Bootstrap values more than 80% are shown at nodes.

Phylogenetic analysis of sequences from 50 different countries during the last 65 years from 1952 to 2017 showed various clustering patterns in association with regions and year of circulation of the strains (Fig. 2, Supplementary Fig. S1 and Table S1). The strains reported from countries like Nigeria, Senegal and Cote D'ivoire during 1964–2009 clustered in West African genotype. The Asian genotype consisted of the strains reported from India during 1963–2000 and the sequences from 1962 to 2017 from Thailand, Philippines, Indonesia, Malaysia and Micronesia. Recently reported strains from 2014 to 2017 from the Caribbean region, Columbia, Brazil and Mexico clustered in the Asian clade and were found similar to the strains circulating in Indonesia, China and the Philippines. The ECSA genotype is geographically more dominant and widely spread across the world. These include various countries in South, central and East Africa. Additionally, the reports of the circulation of these strains to many Asian countries have also been documented to start from the year 2004. However, the ECSA strains have entered the Americas as well as Europe during the recent past.

Further, the analysis of the global CHIKV sequences suggested interesting clustering pattern of the sequences, i.e. three clusters in ECSA (I, II, III) and two clusters (A and B) in the Asian genotype. The clustering patterns have been described in detail in the Discussion section for both the phylogenetic and MCC trees.

### Phylogenetic networking

**Fig. 3.**
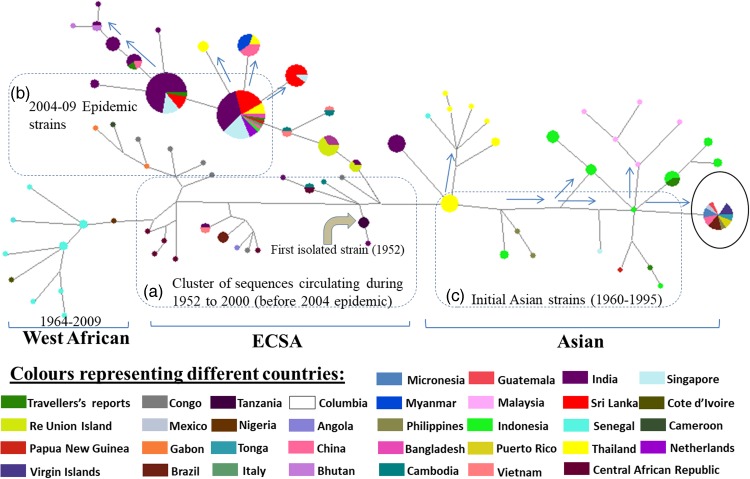
The phylogenetic Network showing clusters of the Chikungunya virus sequences from different geographical regions. The network shows the pattern of emergence of CHIKV related to the first isolated strains. The size of the circles is representative of the number of clustering haplotypes from different parts of the world. The sequences in panels a, b and c show probable origin of the virus in ECSA strains, the distinct epidemic strains and the initial Asian strains. The cluster highlighted with a black circle contains recently circulating strains of Brazil, Mexico, the Caribbean and Micronesia. The arrows show the divergence of new and recently reported strains from their origin. The length of lines and distances between strains are not proportional to the mutational distances amongst these strains.

The phylogenetic network showed distinct branching of all the three genotypes (Fig. 3, Supplementary Fig. S2 and Table S1). All the CHIKV strains formed a network that is illustrated in panels a, b and c. Panel ‘a’ contains CHIKV strains reported from Tanzania in 1952 (first reported strain) and subsequent strains from Uganda, Central African Republic, Democratic Republic of Congo, Gabon, Tonga, Angola and Cameroon during1960s and 1970s. The Yawat strain happens to be the first isolate of ECSA genotype in India converged directly in the same group. The West African strains from Nigeria (1966), Cote d'ivoire (1981) and Senegal (1966) deviated slowly to branch out from these initial strains.

The 2004–09 epidemic strains circulated in most of the Indian Ocean regions and other countries like India, Sri Lanka, Bangladesh, France, Italy, China, Singapore, the Netherlands and Reunion Islands. All these strains clustered in panel ‘b’ and diverged from the initial strains of Tanzania and the Central African Republic reported during 1952–56 (ECSA genotype). The recently circulating CHIKV in countries like India, Bhutan, Vietnam, Cambodia, Myanmar, Thailand, USA, Mexico and Brazil were found to deviate from 2004–09 epidemic strains.

The third panel ‘c’ included the sequences of the Asian genotype from India, Indonesia and Thailand. These strains included sequences from India before 2004 and sequences from the 1960s till the latest available strains of 2017 from other Asian countries such as Indonesia, Malaysia, Philippines and Thailand. Several recently reported CHIKV strains (2012–17) from Brazil, Mexico, Puerto Rico, Columbia, Guatemala, Virgin Islands, New Caledonia, Papua New Guinea and Micronesia also diverged from this group.

### Bayesian evolutionary analysis

**Fig. 4.**
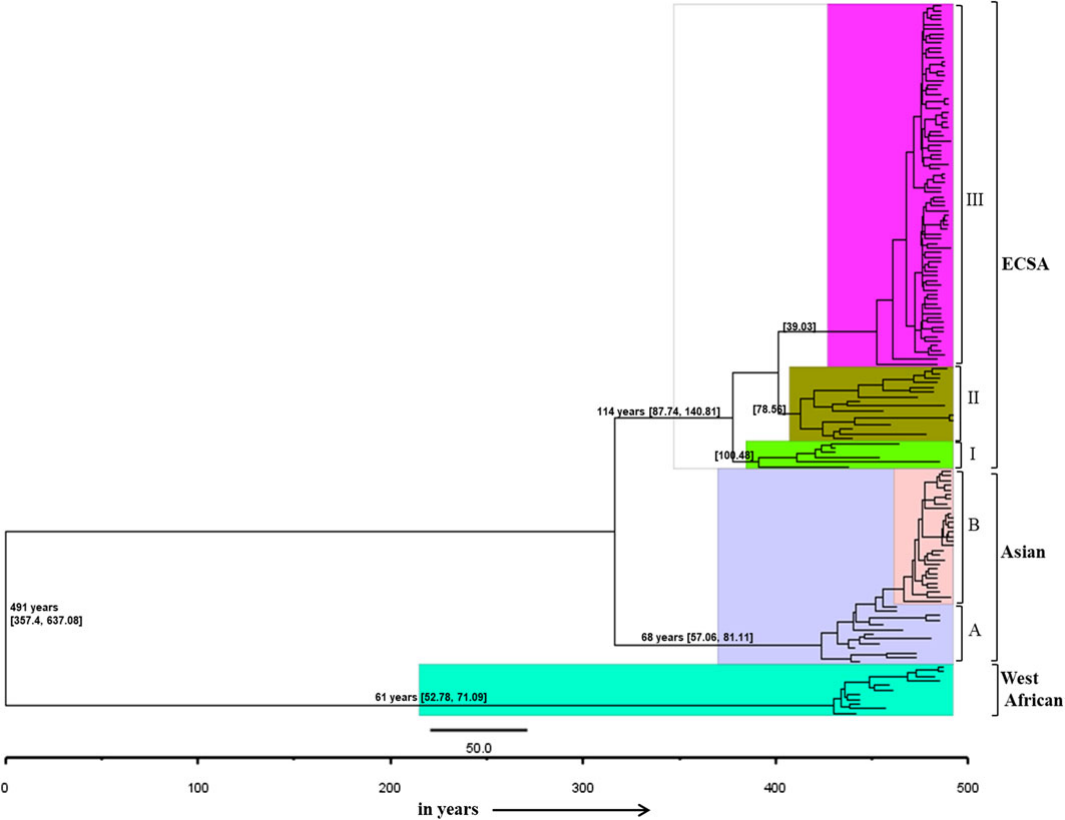
The MCMC Bayesian tree generated using different Chikungunya virus strains from various parts of the world. The age is mentioned on major branches with HPD 95% height.

Bayesian skyline coalescent tree prior under strict clock was chosen to be the best fit on the basis of Bayes factor (Supplementary Table S2). The Maximum Clade Credibility Tree was constructed using the log file generated in the best run (Fig. 4). The MCMC tree also revealed the clustering pattern of the sequences as described earlier by phylogenetic analysis. The CHIKV was calculated to be originated around 491 years ago (1526) with 95% Highest Posterior Density (HPD) ranging from 357 (1660) to 637 years (1380) with a global nucleotide substitution rate of 2.78 × 10^−4^ (HPD 95%: 2.14 × 10^−4^ to 3.47 × 10^−4^) across all the genotypes. The tMRCA of ECSA was calculated to be 114 years (1903) (HPD 95% 87 (1930) to 140 (1877)). The age of Asian and West African genotypes was calculated to be 68 years (1949) (HPD 95% 57 (1960) to 81(1936)) and 61 years (1956) (HPD 95% 52 (1965) to 71 (1946)), respectively.

**Fig. 5.**
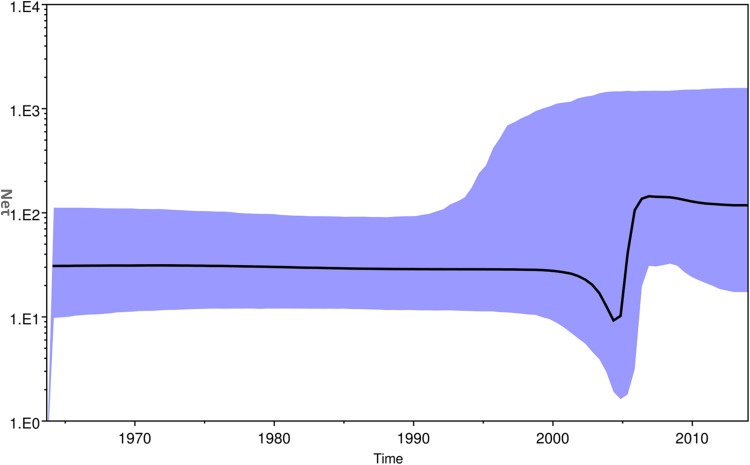
The Bayesian skyline plot. The plot shows viral population *vs.* time of circulation of the Chikungunya virus strains from India.

The Bayesian skyline plot of Indian strains from 1963 to 2017 was generated for the product of effective population size and generation time (Ne*τ*) *vs.* time (Fig. 5) [30]. The plot showed almost a constant phase of CHIKV infection till 2000 and then a slight decrease. Following this, a sudden increase in the virus population was attributed to the 2004–09 epidemics. Subsequently, almost stable virus population was reported during 2010–2017.

### Shannon entropy analysis

The Shannon entropy analysis of the sequences showed variable sites among the unique global sequences (Supplementary Fig. S3). The entropy value above the threshold of 0.2 is indicative of variations at these sites. A total of 12 variable sites were selected at the amino acid positions 211, 225, 226, 269, 276, 284, 304, 321, 343, 344, 397 and 404 with entropy values higher than 0.2. The positions numbers 211, 226, 269 and 284 had high entropy values (≥0.8).

### Selection pressure analysis

The results of selection pressure analysis are summarised in Table 1 with normalised dN/dS. The low dN/dS ratio (range is 0.143–0.187) suggested purifying selection in this region of the E1 gene. Nine amino acids were found to be positively selected by FEL (211, 226,291,297,304, 321, 344, 374 and 377). Seven positively selected positions (211, 269, 291, 304, 321, 374 and 377) were identified by IFEL method and four by SLAC method (211, 226, 304 and 321). Three amino acid positions (211, 304 and 321) were found to be under positive selection by all the three methods and two amino acids (226 and 377) by two different methods. Significantly, a mutation at 211 position was identified in most of the strains of all the three genotypes. Some of the mutations were identified to be genotype specific. We observed that three of the codon positions at 225, 304 and 377 mutated in most of the Asian genotypes, whereas the mutations at 226 and 284 positions were identified in most of the ECSA strains.

**Table 1.** Selection pressure analysis of the E1 gene of the Chikungunya virus. The details of positively selected sites under different substitution model and methods are summarized in the table

*P* value	Nucleotide substitution methods											
	SLAC						FEL			IFEL		
	F81		HKY85		REV		F81	HKY85	REV	F81	HKY85	REV
	dN/dS	+ ve sites	dN/dS	+ ve sites	dN/dS	+ ve sites	+ ve sites	+ ve sites	+ ve sites	+ ve sites	+ ve sites	+ ve sites
0.1	0.143	N	0.186	N	0.187	N	304, 321, 377	304, 321	304, 321	304, 321	304, 321	304, 321
0.15	0.143	304	0.186	304	0.187	304	304,321, 344,377	304, 321, 377	304, 321, 377	304, 321	304, 321	304, 321
0.2	0.143	304, 321	0.186	304, 321	0.187	304, 321	226, 291, 297,304, 321, 344, 374, 377	211, 226, 304, 321, 377	304, 321, 377	304, 321	211, 304, 321	304, 321
0.25	0.143	226, 304, 321	0.186	211, 226, 304, 321	0.187	226, 304, 321	226,291, 297,304, 321, 344, 374, 377	226, 297,304, 321, 344, 374, 377	211, 304, 321, 377	269,291, 304,321, 374, 377	211, 304, 321, 377	211, 304, 321 377

## Epitope mapping

### Identification of B-cell epitopes

Six different prediction methods available in IEDB were used to predict the probable B-cell epitopes (Supplementary Table S3, Supplementary Fig. S4). This included the following: (1) Bepipred linear epitope prediction: the peptide ranging from amino acid number 185 to 214 had higher prediction values with a maximum score of 1.8. The average and minimum scores were 0.089 and −3.2, respectively, with a threshold value of 0.09. (2) Chou and Fasman *β*-turn prediction: amino acid number 180–215 with a higher prediction score of 1.25 and an average and minimum score of 0.9 and 0.5, respectively, with a threshold value of 1.00. (3) Emini surface accessibility prediction: maximum surface accessibility was found in the region from 200 to 215 amino acids with a score of 6.0 with a minimum and average surface accessibility score of 0.03 and 1.00, respectively, at a threshold value of 1. 4. The highest flexibility score calculated by Karplus and Schulz prediction was 1.095 in the 190–212 amino acids with an average of 0.9 and a minimum score of 0.8 at a threshold value of 1. (5) Kolaskar and Tongaonkar method: higher score of 1.2 amino acids 210–220 with an average, minimum and maximum scores of 1.056, 0.9 and 1.3 at a threshold value of 1. (6) Parker Hydrophilicity Prediction calculated the highest value of 5.9 in 200–220 amino acid positions with an average score of 1.46 and a minimum score of −7.2 at a threshold of 1.48. Amino acid positions from 200 to 214 were found to be common by almost all the prediction methods.

### Identification of T-cell epitopes

A total of 31 potential epitopes were shortlisted in the E1 protein of CHIKV and were analysed in subsequent steps. Three peptides (YPFMWGGAY, KVFTGVYPF and FMWGGAYCF) were finally selected on the basis of all the parameters summarised in Supplementary Table S4.

The potential epitopes for MHC-II in the E1 protein of CHIKV were initially predicted in IEDB peptide binding to MHC-II molecules. A total of 45 potential epitopes for HLA-DRB1 were further investigated in PREDIVAC online tool. On the basis of high predivac score and IEDB analysis, four peptides were predicted to be the potential epitopes (VHSMTNAVT, WLKERGASL, IKYAVSKKG and YKTLVNRPG) and the details of scores and associated alleles are summarised in Supplementary Table S5.

### Docking of selected potential epitopes

**Fig. 6.**
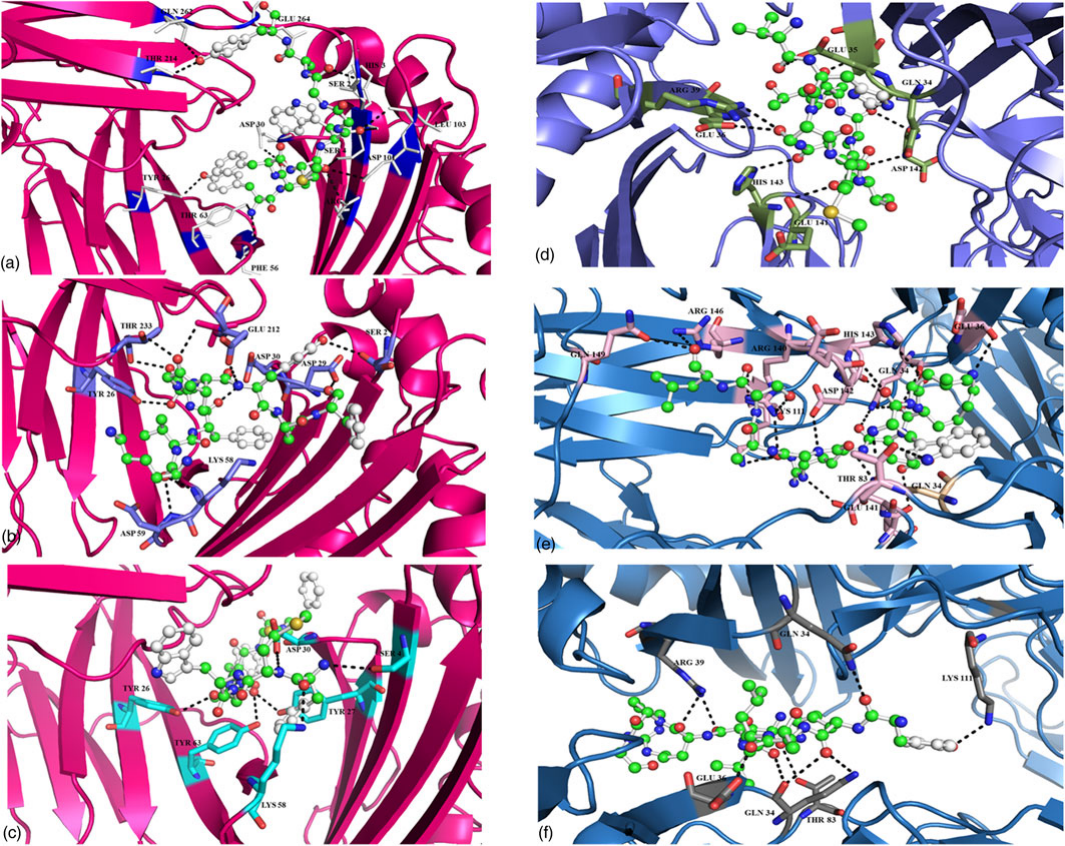
Docking of the potential epitopes of the E1 protein of the Chikungunya virus with MHCI/II alleles. The images a, b and c show the interaction of HLA-A*35:01 with the three selected epitopes for MHCI molecules (details in the Supplementary Table S4). The images d, e and f show the interaction of HLA-DRB0101 with the first, second and fourth epitopes selected for MHCII molecules (details in the Supplementary Table S5).

The six shortlisted epitopes out of seven were docked with the alleles found common for them for MHCI and MHCII molecules. The alleles used for the docking of the MHCI molecules were HLA-A*35:01 and HLA-DRB0101 for MHCII molecules. One peptide ‘IKYAVSKKG’ was not docked due to the unavailability of the tertiary structure of HLA-DRB0806. All the epitopes were found to have a very good interaction with their respective alleles and exhibit high binding energies (Fig. 6). The details of docking with the main interacting residues and respective binding energies are summarised in Table 2.

**Table 2.** Docking scores of the shortlisted alleles. The docking scores of different alleles and their corresponding interacting residues of all the six epitopes

Epitope sequence and docked allele	Binding energy (kcal/mol)	Interacting residue
YPFMWGGAY with HLA-A*35:01	−8.7	TYR 26, TYR 63, PHE 56, ARG 6, ASP 102, LEU 103, SER 4, HIS 3, SER 2, ASP 30, GLU 264, THR 214, GLN 262
KVFTGVYPF with HLA-A*35:01	−8.3	ASP 59, LYS 58, ASP 29, SER 2, ASP 30, GLU 212, THR 233, TYR 26
FMWGGAYCF with HLA-A*35:01	−9.5	SER 4, TYR 27, ASP 30, LYS 58, TYR 63, TYR 26
VHSMTNAVT with HLA-DRB0101	−7.4	GLU 35, GLN 34, GLU 36, ASP 142, HIS 143, GLU 141, ARG 39
WLKERGASL with HLA-DRB0101	−8.2	GLU 141, ASP 142, GLN 34, THR 83, GLN 34, GLU 36, ASP 142, HIS 143, LYS 111, ARG 140, ARG 146, GLN 149
YKTLVNRPG with HLA-DRB0101	−7.5	THR 83, LYS 111, GLN 34, GLU 36, ARG 39

## Discussion

CHIKV has been described from different geographical regions including autochthonous transmission and travel-associated cases. A few reports also relate to the evolutionary divergence of CHIKV in different parts of the world [1, 2, 31]. Outbreaks of Chikungunya fever across the globe, spread of infection across a population and overall mutational load are the three main variables of the transmission. These variables may operate independently or collectively to give rise to a particular pattern of infection. The present study has attempted to gain an insight into the organisation, distribution and mutational load of the virus strains reported from different parts of the world. We evaluated the transmission dynamics of the CHIKV across the globe using phylogenetic, Network and Bayesian analysis focusing on all the three genotypes comprising West African, Asia and ECSA. The sequences were used from 50 different countries that showed well-established autochthonous transmission and some travel-associated cases from 1952 to 2017 encompassing 65 years. We undertook genetic characterisation of the viral strains by mutational analysis, variable sites, entropy, selection pressure and epitope mapping.

The evidence of CHIKV occurrence in different geographical regions was reported as early as in the 1770s that was suggested by Carey in 1971 [32]. In 1779, Batavia recorded knuckle (joints) fever, in Cairo named as knee trouble, scarletina rheumatic in Calcutta, Madras and Gujarat in 1824–25 [32]. Our data also supported that the CHIKV infection in humans originated around 491 years ago (1526). This tMRCA is similar to the reports that described the origin of CHIKV to be of 300–500 years age [1, 2]. The origin of the ECSA genotype was reported to be around 114 years ago (1903). Initially, this genotype was confined to the central, East and South African regions. Later on, the CHIKV diverged from these initial strains and were transmitted to other geographical regions that constituted the West African and Asian genotypes. The West African genotype originated from the Central African region around 61 years ago (1956). Whereas, the Asian strains descended 68 years ago (1949). The substitution rate was found to be 2.78 × 10^−4^ for the partial E1 gene compatible with other studies reporting 2.3 × 10^−4^ to 4.6 × 10^−4^ for the complete E1 gene [2].

The phylogenetic and Bayesian analyses of the global CHIKV sequences revealed interesting clustering of the sequences. Both the analyses suggested a similar branching pattern thereby delineating the spread of CHIKV in different geographic regions. The ECSA strains formed three clusters (I, II and III) whereas the Asian sequences formed two clusters (A and B). Cluster I consisted of the initial ECSA strains that were present in the African region during the 1950s and 1960s. Cluster II consisted of sequences from the African region that were circulating after the year 2000. Cluster III included all the strains of the 2004–09 epidemic in the Indian Ocean region including the IOL strains (Indian Ocean Lineage). In addition, this cluster also included the currently circulating ECSA strains of the Asian and American regions. Likewise, the Asian clade was sub-grouped into two clusters A and B. Cluster A grouped all the strains that circulated initially in the Asian region during the 1960s and 1970s. Group B included the strains that are recently circulating in regions like Papua New Guinea, Indonesia, etc. Further, some strains circulating in the Americas and Caribbean region also clustered in this group.

The Network analysis revealed that in the West African region, Nigerian CHIKV strain further diverged to Senegal and Cote D'ivoire [2, 33]. This West African genotype was localised in this region from the late 1950s till 2009 that was not reported from other geographical regions. Our data also suggested that the emergence of the Asian genotype was from Thailand due to the first Asian epidemic in this region during 1958 [34, 35]. Subsequently, the Asian genotype from Thailand diverged in three different directions. The first branch spread towards India, the second one continued circulating in Thailand and the third one moved to Indonesia, Malaysia and the Philippines. These strains in India and Thailand did not branch further and were observed until the 2004 epidemic. The third branch further diverged towards the Pacific Ocean and the Caribbean region. We further analysed the divergence of this third branch of the Asian genotype in detail. The epidemic involving Asian strains started from Indonesia in 2010 causing the first outbreak in New Caledonia in 2011, Philippines, Yap Island (Micronesia) and China in 2012 [36, 37]. This branch further moved towards other Pacific Ocean regions and French territories including Tonga and Papua New Guinea. Similar strains were also reported from East Timor, Moscow, France and the Caribbean Islands [37]. The travel-associated spread of the virus in some areas of the Caribbean region lead to the establishment of mosquito–human life cycle of the virus in these areas. Subsequently, many autochthonous cases were reported from these regions [9, 36]. The virus then spread throughout the Caribbean region and then to Brazil, Mexico and French Polynesia [9, 38–40].

The Asian genotype of CHIKV was reported to have caused epidemics in many Asian countries from 1958 to 1973, subsequently followed by the reports of sporadic cases. Subsequently, the genotypic shift and introduction of ECSA strains in the Asian countries were initiated during 2004 [41]. CHIKV initially circulated in major parts of Africa till 2004. The infection later on spilled over to the Indian Ocean region and Indian subcontinent leading to the epidemics in this region. The Eastward movement of ECSA strains was observed from Kenya to Comoros, Reunion Island covering the Indian subcontinent and subsequently to other Asian countries [42]. This progressive spread of CHIKV initiated an explosive epidemic that affected millions of inhabitants in this region including India during 2005–2009 [11]. These epidemic strains branched independently from the node of initial ECSA strains and formed a separate branch known as IOL [43]. Subsequently, these strains spread to European countries including France, Italy and the Netherlands through travellers. This resulted in small outbreaks of autochthonous cases in these European countries [44]. Thus, the ECSA strains were reported from various geographical regions confirming its global spread.

The ECSA strains are presently circulating in many Asian countries namely India, Sri Lanka, Bhutan, China, Singapore, Cambodia and Vietnam that originated from a cluster of the epidemic strains [45–49]. The other branch of the epidemic strains diverged into the sequences reported in recent years from Thailand, Sri Lanka and American regions (including Brazil, Mexico and USA). Thus, during recent years (2013–14), ECSA has started a parallel circulation with Asian strains in the American continent and the Caribbean regions [9]. Further, the phylogenetic analysis of the ECSA genotype suggested a close similarity between these 2013–17 strains reported from the USA and one particular travel-associated case that was imported from Angola to the USA [9]. These branching patterns of CHIKV strains across the globe based on the E1 gene reflect its gradual and consistent evolution.

A detailed genetic characterisation of the global CHIKV strains was done using the variable sites, selection pressure and epitope mapping of the E1 protein. The neutral selection pressure, positively selected and variable sites have been determined for all the genotypes suggesting the stochastic process of evolution. Four of amino acid positions 211, 226, 304 and 321 were positively selected and had high entropy suggesting higher chances of mutation at these sites. The mutation at A226V was positively selected, had high entropy and was previously associated with the increased epidemic potential of the virus by enhancing the efficiency of mosquito transmission during 2004–09. The molecular along with the epidemiological studies can help in determining the importance of accumulation of these adaptive mutations in the selected genotypes/strains which might further affect the transmission of the virus to new territories [19].

It may be noted that the present study describes the first report of detailed B- and T-cell epitope mapping of the E1 protein of CHIKV. All the selected potential peptides were present on the surface of the E1 protein. In addition, the peptides selected as likely epitopes for MHC-II molecules were present in domains I and III of the E1 protein. Domains I, II and III of the E1 protein were described in the crystal structure of the structural polyproteins of CHIKV [50]. The T-cell epitopes induce long-lasting cell-mediated innate immune responses and thus vaccines against T-cell epitopes are considered to be most promising [51]. These *in silico* results need to be validated by future research. Taken together, the computational and experimental authentication of epitope mapping might contribute towards the formulation of effective vaccine strategies against this re-emerging viral pathogen.

## Conclusions

The reconstruction of the geographic distribution of CHIKV suggested a distinctive branching pattern owing to gradual alterations in the E1 gene. The recent movement of the virus to non-endemic regions of the world is associated with travel cases and its autochthonous transmission. Co-circulation of CHIKV with other arthropod-borne viruses like Dengue and Zika in many regions has been reported from different geographical regions. Overlapping clinical symptoms and limited diagnostic tests, particularly in underdeveloped countries, can complicate the accurate reporting of CHIKV infections. Therefore, a comprehensive surveillance of all the arthropod-borne viruses at both hospital and community levels would go a long way in determining the transmission dynamics of these pathogens. This is likely to provide intellectual enrichment on the global disease burden and the formulation of possible control measures for the prevention of these viral infections.

## Supplementary material

For supplementary material accompanying this paper visit https://doi.org/10.1017/S0950268820000497. click here to view supplementary material
