# Supplementary material for: Global transmission and evolutionary dynamics of the Chikungunya virus
Source: Epidemiol Infect. 2020 Feb 19;148:e63. doi: 10.1017/S0950268820000497 (PMC7118414; doi:10.1017/S0950268820000497)
Supplement: Supplementary file 1 [file S0950268820000497sup001.zip › supplimentary figures.pdf]

Global transmission and evolutionary dynamics of the Chikungunya virus

F. Deeba[1], M. S. H. Haider[1], A. Ahmed[2, 3], A. Tazeen[1], M. I. Faizan[1], N. Salam[4], T. Hussain[3], S. F. Alamery[3], S. Parveen[1]*

1. Centre for Interdisciplinary Research in Basic Sciences, Jamia Millia Islamia, New Delhi, India

2. Protein Research Chair, Department of Biochemistry, College of Science, King Saud University, Riyadh, Saudi Arabia

3. Centre of Excellence in Biotechnology Research, College of Science, King Saud University, Riyadh, Saudi Arabia

4. Department of Pathology, College of Medicine, Al-Imam Mohammad Ibn Saud Islamic University (IMSIU), Riyadh, Saudi Arabia

*Corresponding author

Shama Parveen, Centre for Interdisciplinary Research in Basic Sciences, Jamia Millia Islamia, New Delhi- 110025, INDIA

E-mail: shamp25@yahoo.com, sparveen2@jmi.ac.in

Supplementary figures: 4 (S1-S4)

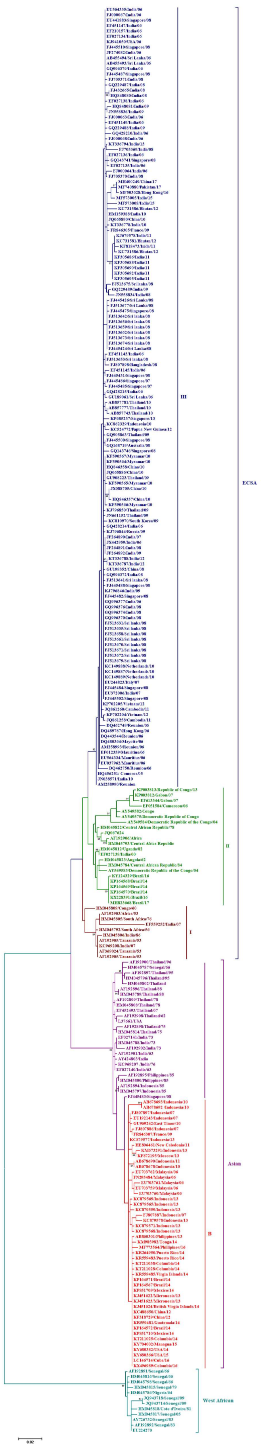
III
ECSA
II
I
Asian
West African

Supplementary figure S1: The detailed version of ML phylogenetic tree of Chikungunya virus. The bootstrap values above 80% are displayed on the nodes. The strains have been represented by their respective accession number followed by country and year of isolation.

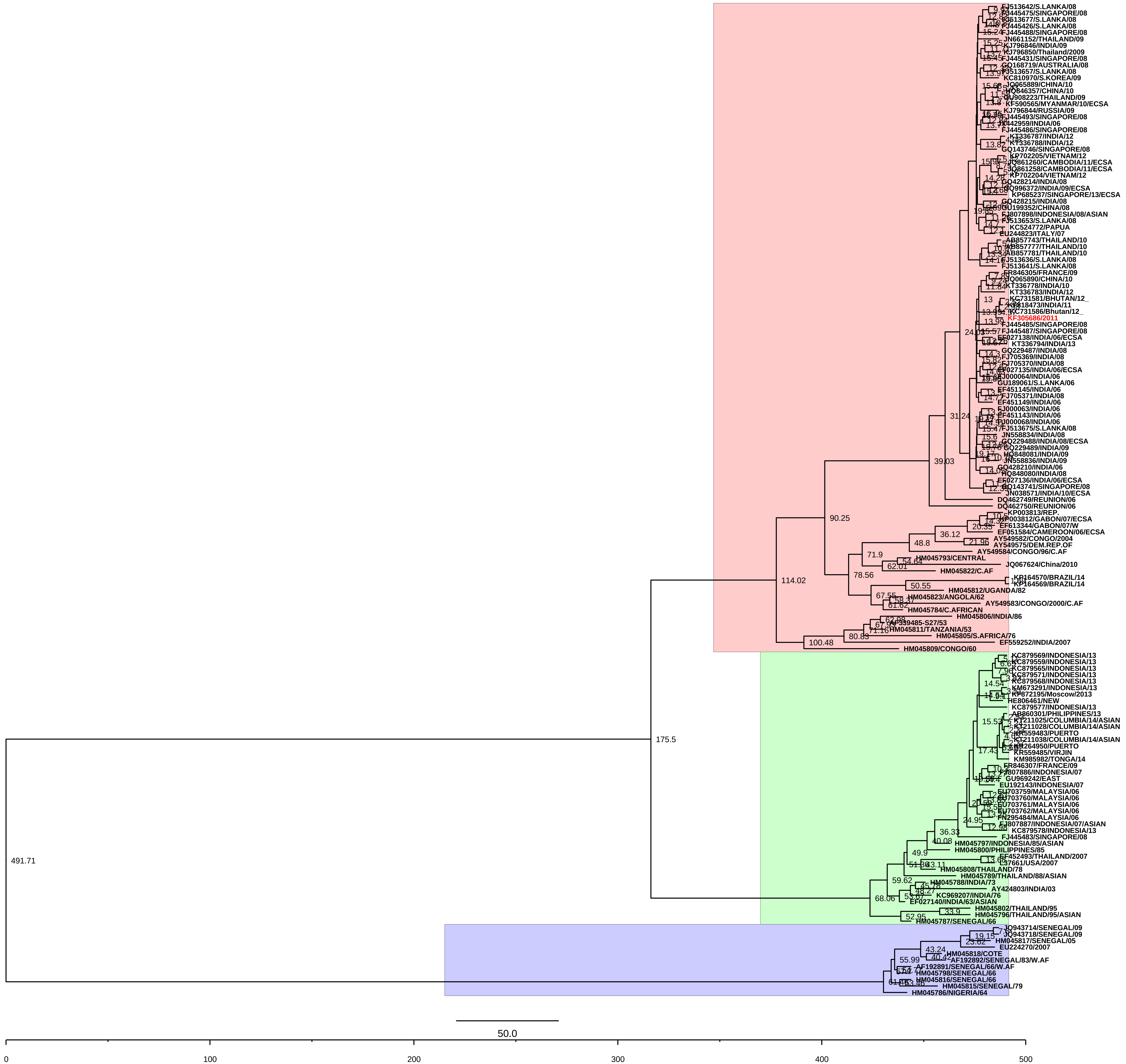

FJ513642/S.LANKA/08
SINGAPORE/08
S.LANKA/08
FJ445426/S.LANKA/08
FJ445488/SINGAPORE/08
JN661152/THAILAND/09
KJ796846/INDIA/09
KJ796850/Thailand/2009
FJ445431/SINGAPORE/08
GQ168719/AUSTRALIA/08
FJ513657/S.LANKA/08
KC810970/S.KOREA/09
JQ065889/CHINA/10
HQ846357/CHINA/10
GU908223/THAILAND/09
KF590565/MYANMAR/10/ECSA
KJ796844/RUSSIA/09
FJ445493/SINGAPORE/08
EU442959/INDIA/06
FJ445486/SINGAPORE/08
KT336787/INDIA/12
KT336788/INDIA/12
GQ143746/SINGAPORE/08
KP702205/VIETNAM/12
JQ861260/CAMBODIA/11/ECSA
JQ861258/CAMBODIA/11/ECSA
KP702204/VIETNAM/12
GQ428214/INDIA/08
JQ996372/INDIA/09/ECSA
KP685237/SINGAPORE/13/ECSA
GQ428215/INDIA/08
GU199352/CHINA/08
FJ807898/INDONESIA/08/ASIAN
FJ513653/S.LANKA/08
KC524772/PAPUA
EU244823/ITALY/07
AB857743/THAILAND/10
AB857777/THAILAND/10
AB857781/THAILAND/10
FJ513636/S.LANKA/08
FJ513641/S.LANKA/08
FR846305/FRANCE/09
JQ065890/CHINA/10
KT336778/INDIA/10
KT336783/INDIA/12
KC731581/BHUTAN/12_
INDIA/11
KC731586/Bhutan/12_
KF305686/2011
FJ445485/SINGAPORE/08
FJ445487/SINGAPORE/08
EF027138/INDIA/06/ECSA
KT336794/INDIA/13
GQ229487/INDIA/08
FJ705369/INDIA/08
FJ705370/INDIA/08
EF027135/INDIA/06/ECSA
FJ000064/INDIA/06
GU189061/S.LANKA/06
EF451145/INDIA/06
FJ705371/INDIA/08
EF451149/INDIA/06
FJ000063/INDIA/06
EF451143/INDIA/06
FJ000068/INDIA/06
FJ513675/S.LANKA/08
JN558834/INDIA/08
GQ229488/INDIA/08/ECSA
GQ229489/INDIA/09
HQ848081/INDIA/09
JN558836/INDIA/09
GQ428210/INDIA/06
HQ848080/INDIA/08
EF027136/INDIA/06/ECSA
GQ143741/SINGAPORE/08
JN038571/INDIA/10/ECSA
DQ462749/REUNION/06
DQ462750/REUNION/06
KP003813/REP.
KP003812/GABON/07/ECSA
EF613344/GABON/07/W
EF051584/CAMEROON/06/ECSA
AY549582/CONGO/2004
AY549575/DEM.REP.OF
AY549584/CONGO/96/C.AF
HM045793/CENTRAL
JQ067624/China/2010
HM045822/C.AF
KP164570/BRAZIL/14
KP164569/BRAZIL/14
HM045812/UGANDA/82
HM045823/ANGOLA/62
AY549583/CONGO/2000/C.AF
HM045784/C.AFRICAN
HM045806/INDIA/86
AF339485-S27/53
HM045811/TANZANIA/53
HM045805/S.AFRICA/76
EF559252/INDIA/2007
HM045809/CONGO/60
KC879569/INDONESIA/13
KC879559/INDONESIA/13
KC879565/INDONESIA/13
KC879571/INDONESIA/13
KC879568/INDONESIA/13
KM673291/INDONESIA/13
KP872195/Moscow/2013
HE806461/NEW
KC879577/INDONESIA/13
AB860301/PHILIPPINES/13
KT211025/COLUMBIA/14/ASIAN
KT211028/COLUMBIA/14/ASIAN
KR559483/PUERTO
KT211038/COLUMBIA/14/ASIAN
KR264950/PUERTO
KR559485/VIRJIN
KM985982/TONGA/14
FR846307/FRANCE/09
FJ807886/INDONESIA/07
GU969242/EAST
EU192143/INDONESIA/07
EU703759/MALAYSIA/06
EU703760/MALAYSIA/06
EU703761/MALAYSIA/06
EU703762/MALAYSIA/06
FN295484/MALAYSIA/06
FJ807887/INDONESIA/07/ASIAN
KC879578/INDONESIA/13
FJ445483/SINGAPORE/08
HM045797/INDONESIA/85/ASIAN
HM045800/PHILIPPINES/85
EF452493/THAILAND/2007
L37661/USA/2007
HM045808/THAILAND/78
HM045789/THAILAND/88/ASIAN
HM045788/INDIA/73
AY424803/INDIA/03
KC969207/INDIA/76
EF027140/INDIA/63/ASIAN
HM045802/THAILAND/95
HM045796/THAILAND/95/ASIAN
HM045787/SENEGAL/66
JQ943714/SENEGAL/09
JQ943718/SENEGAL/09
HM045817/SENEGAL/05
EU224270/2007
HM045818/COTE
AF192892/SENEGAL/83/W.AF
AF192891/SENEGAL/66/W.AF
HM045798/SENEGAL/66
HM045816/SENEGAL/66
HM045815/SENEGAL/79
HM045786/NIGERIA/64
491.71
175.5
114.02
100.48
90.25
80.83
78.56
71.9
68.06
67.55
62.01
61.62
59.62
55.99
54.04
53.67
52.95
50.55
49.9
48.8
43.24
40.08
39.03
36.36
36.33
36.12
33.9
31.24
24.95
24.03
21.96
20.35
17.43
15.53
14.54
13.99
13.68
50.0
0
100
200
300
400
500

Supplementary figure S2: The detailed version of MCMC Bayesian tree generated using different Chikungunya virus strains from various parts of the world. The respective ages are shown on each node. Pink, green and blue regions show the clusters of ECSA, Asian and West African genotypes respectively.

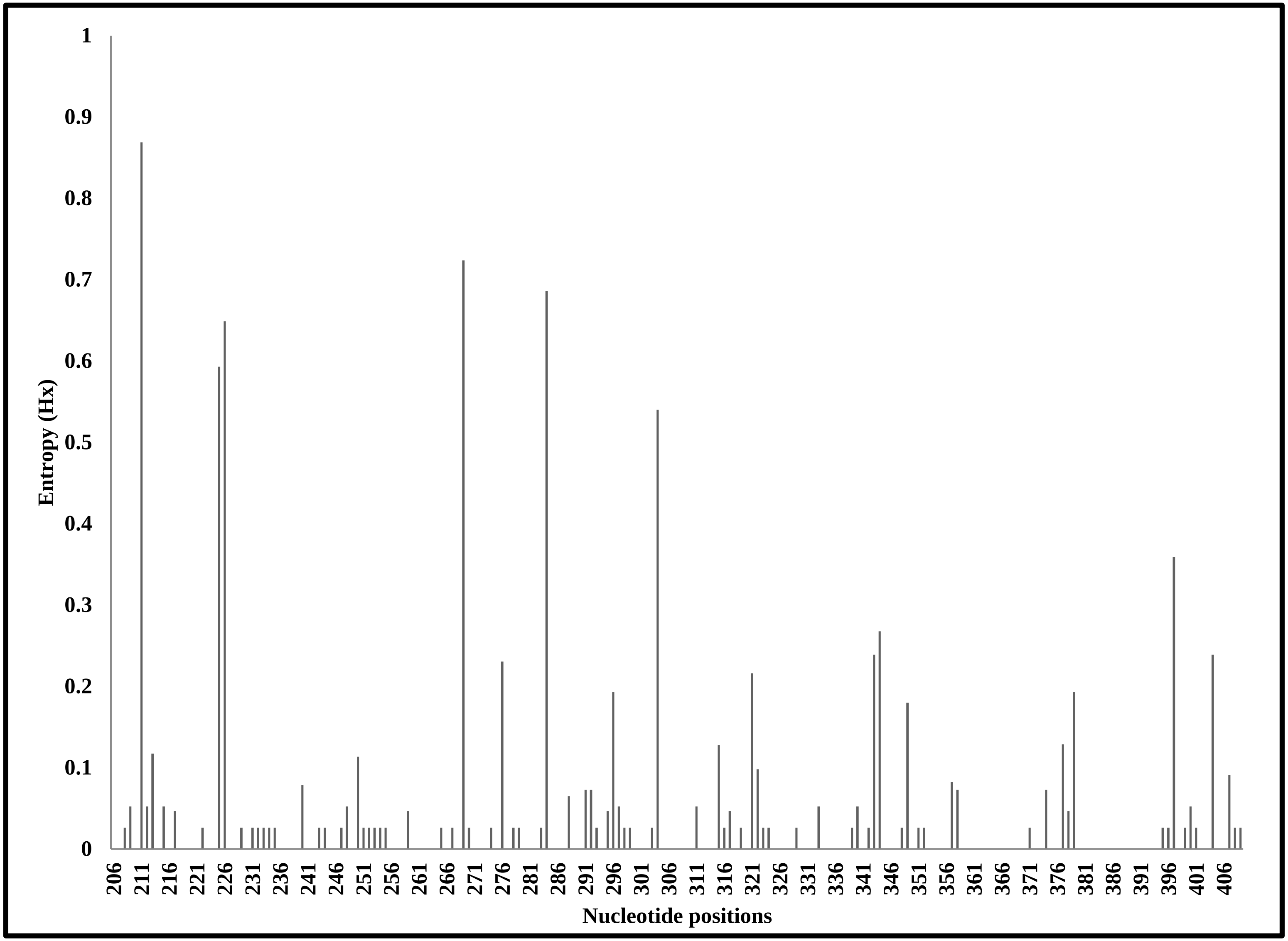

Supplementary figure S3: Shannon Entropy analysis plot showing entropy values of different amino acid residues. The higher entropy value depicts enhanced probability of being a variable site.

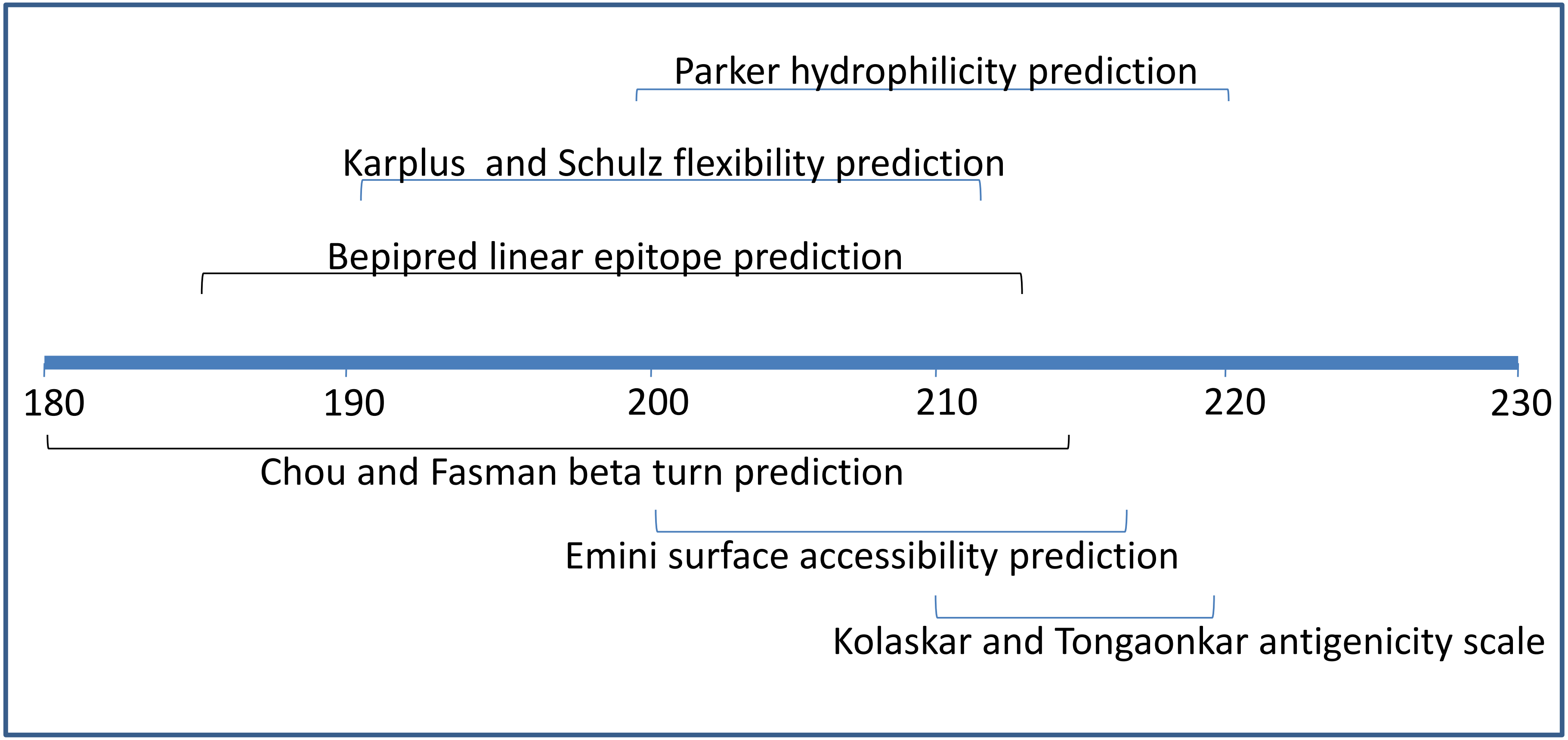

Supplementary figure S4: The amino acid scale showing the most common region between all the B-cell epitope prediction methods having high prediction score.

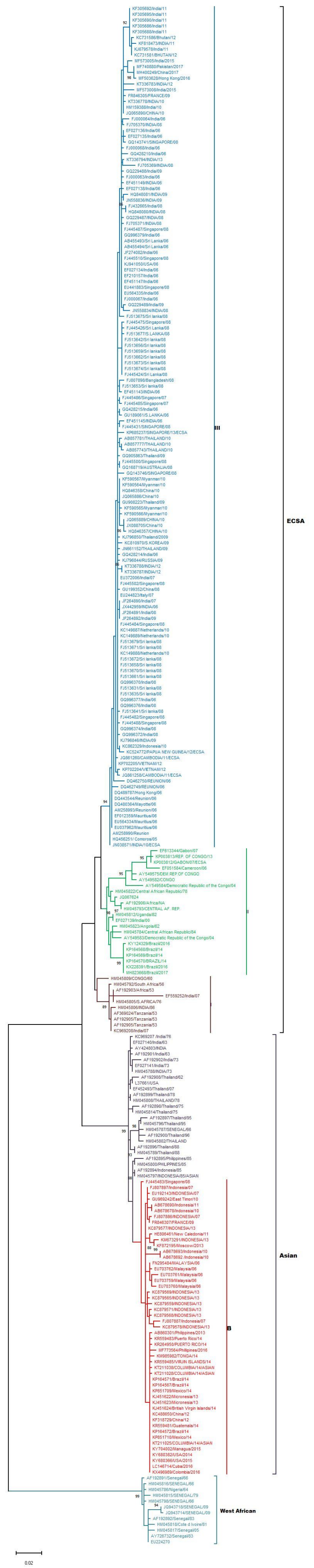

Figure S5: Maximum likelihood tree with thousand bootstrap values generated in Mega X 10.1.5 software
