# Supplementary figures and images for: Global transmission and evolutionary dynamics of the Chikungunya virus
Source: Epidemiol Infect. 2020 Feb 19;148:e63. doi: 10.1017/S0950268820000497 (PMC7118414; doi:10.1017/S0950268820000497)

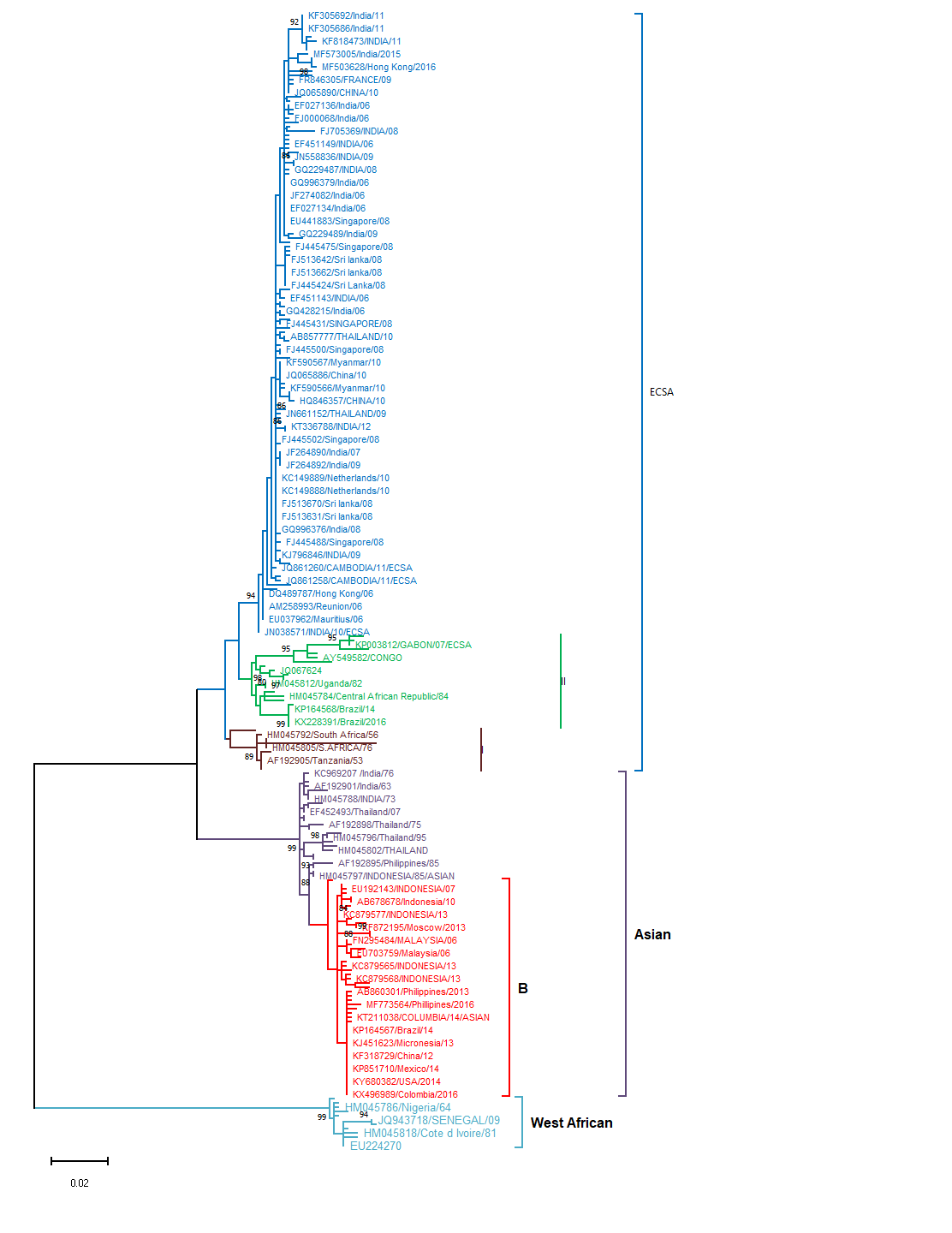

Supplement: Supplementary file 1 [file S0950268820000497sup001.zip › supplimentary figure 5.png]

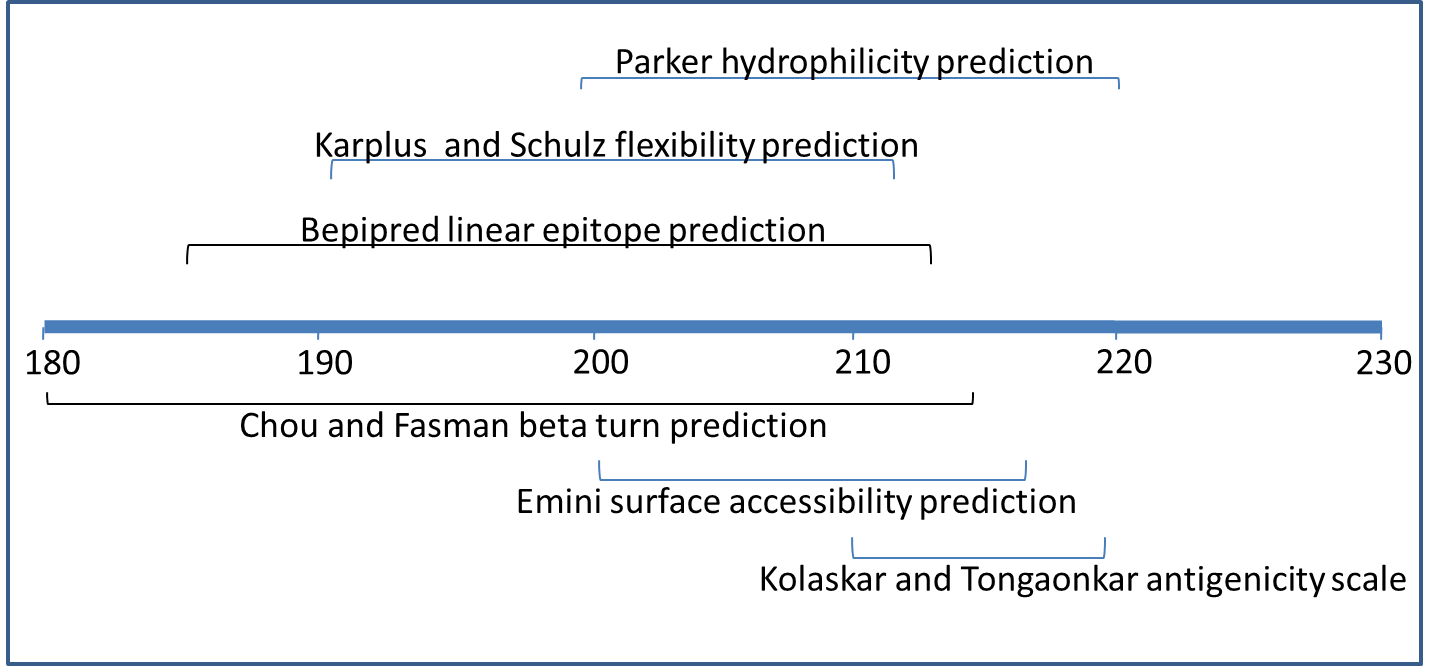

Supplement: Supplementary file 1 [file S0950268820000497sup001.zip › supplimentary figure 4.png]

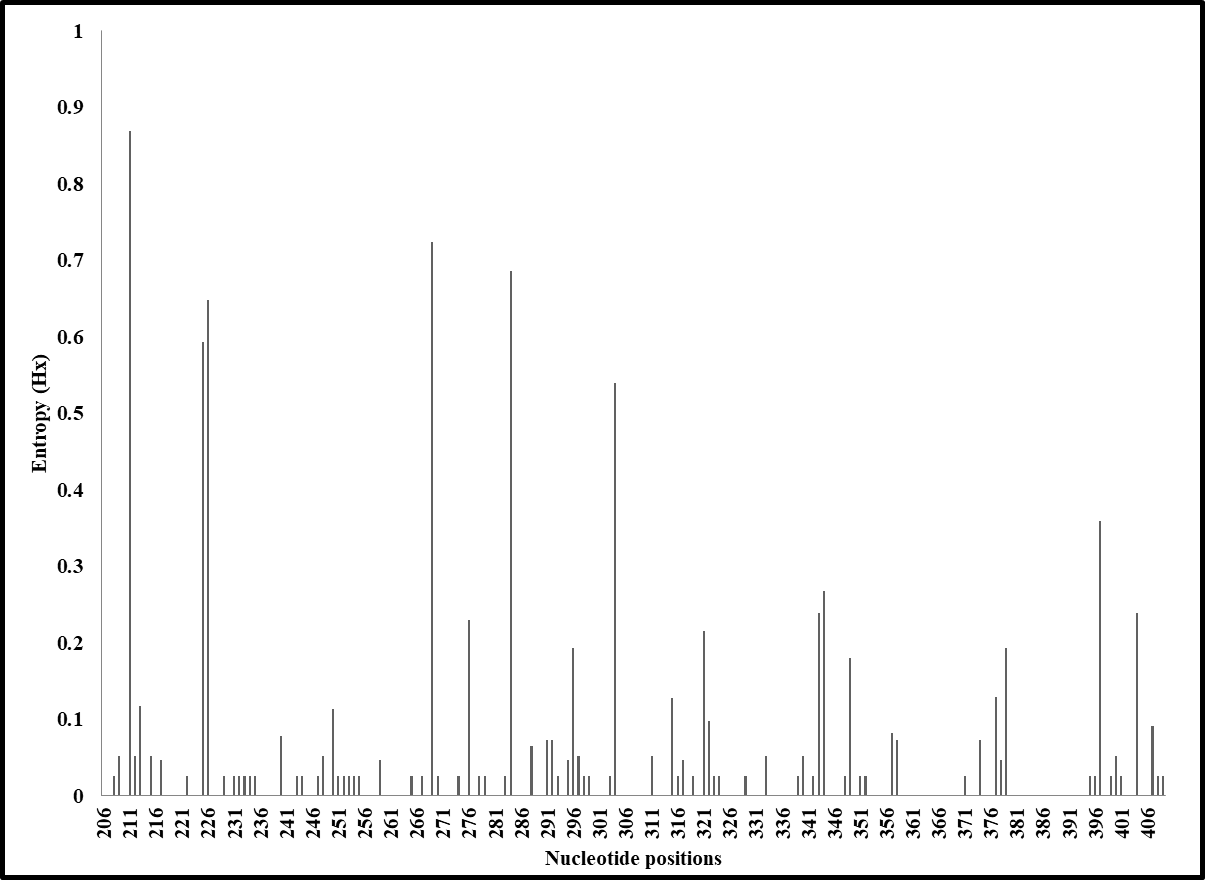

Supplement: Supplementary file 1 [file S0950268820000497sup001.zip › supplimentary figure 3.png]

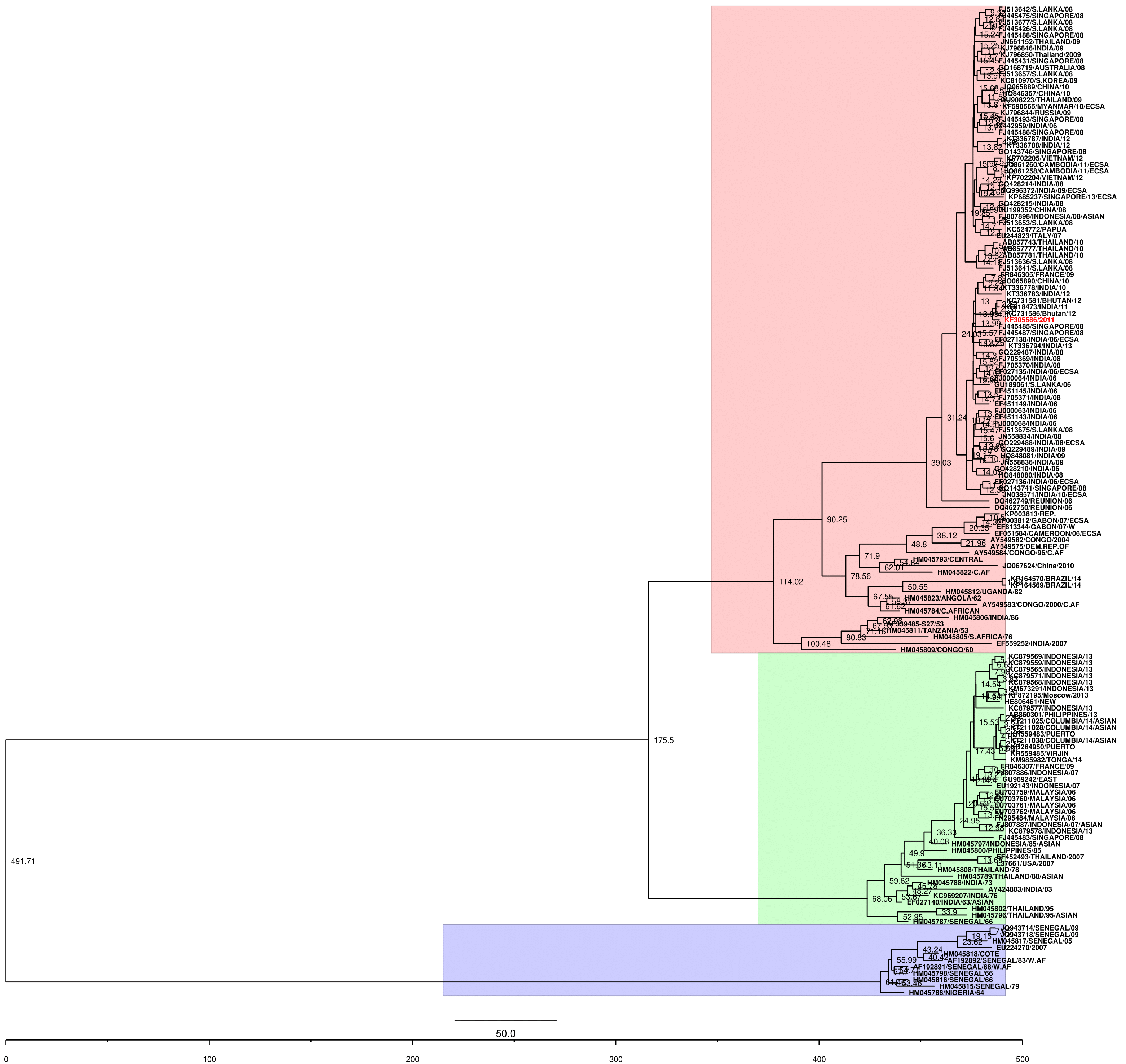

Supplement: Supplementary file 1 [file S0950268820000497sup001.zip › supplimentary figure 2.png]

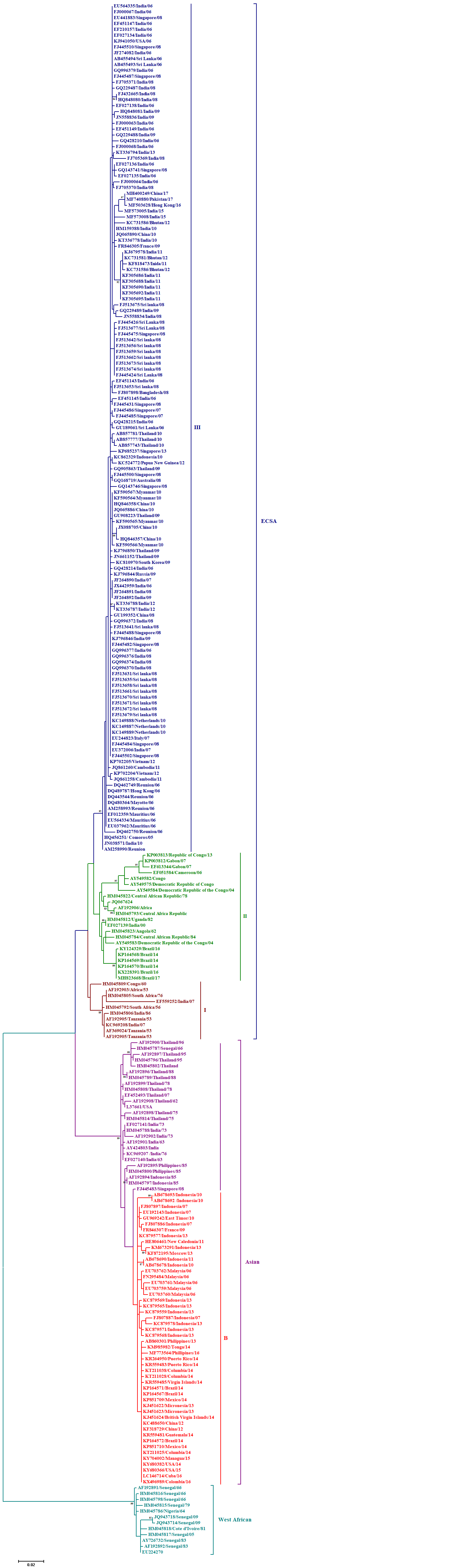

Supplement: Supplementary file 1 [file S0950268820000497sup001.zip › supplimentary figure 1.png]
