# Supplementary material for: Global transmission and evolutionary dynamics of the Chikungunya virus
Source: Epidemiol Infect. 2020 Feb 19;148:e63. doi: 10.1017/S0950268820000497 (PMC7118414; doi:10.1017/S0950268820000497)
Supplement: Supplementary file 1 [file S0950268820000497sup001.zip › supplementary tables.doc]

**Global transmission and evolutionary dynamics of the Chikungunya virus**

Farah Deeba^1^, Md Shakir H. Haider^1^, Ayesha Tazeen^1^, Md I. Faizan^1^, Anwar Ahmed^2^, Nasir Salam^3^, Sher Ali^1^, Zoya Shafat^1^, Ravins Dohare^1^, Irshad H. Naqvi^4^, Shobha Broor^5^, Shama Parveen^1^*

1. Centre for Interdisciplinary Research in Basic Sciences, Jamia Millia Islamia, New Delhi, India

2. Department of Biochemistry, College of Science, King Saud University, Riyadh, Saudi Arabia

3. Department of Pharmacology, College of Medicine, Al-Imam Mohammad Ibn Saud Islamic

University (IMSIU), Riyadh, Saudi Arabia

4. Dr. M. A. Ansari Health Centre, Jamia Millia Islamia, New Delhi, India

5. Department of Microbiology, Faculty of Medicine and Health Science, Shree Guru Gobind

Singh Tricentenary University, Gurgaon, Haryana, India

*Correspondence: E-mail: shamp25@yahoo.com, [sparveen2@jmi.ac.in](mailto:sparveen2@jmi.ac.in)

Supplementary tables: 5 (S1-S5)

**Supplementary Table S1: List CHIKV strains used for phylogenetic analysis, selection pressure, entropy analysis.**

| **GenBank Accession No./ Year of isolation** | **Country** | **Phylogenetic analysis** | **Phylogenetic Networking** | **Bayesian analysis** | **Selection pressure analysis** | **Entropy analysis** |
| --- | --- | --- | --- | --- | --- | --- |
| KF305686/2011 | India | √ | √ | √ | √ | √ |
| KF305687/2011 | India | √ | √ |  |  |  |
| KF305688/2011 | India | √ | √ |  |  |  |
| KF305690/2011 | India | √ | √ |  |  |  |
| KF305692/2011 | India | √ | √ |  |  |  |
| KF305695/2011 | India | √ | √ |  |  |  |
| AB678694/2010 | Indonesia | √ | √ |  |  |  |
| AB678695/2010 | Indonesia | √ | √ |  |  |  |
| AB678693/2010 | Indonesia | √ | √ |  |  |  |
| AB678692/2010 | Indonesia | √ | √ |  |  |  |
| AB678691/2010 | Indonesia | √ | √ |  |  |  |
| AB678690/2011 | Indonesia | √ | √ |  |  |  |
| AB678678/2010 | Indonesia | √ | √ |  |  |  |
| AB678677/2010 | Indonesia | √ | √ |  |  |  |
| AF192908/1962 | Thailand | √ | √ |  |  |  |
| AF192906/ before 1986 | Central African Republic | √ | √ |  |  |  |
| AF192905/1953 | Tanzania | √ | √ |  |  |  |
| AF192903/1976 | Central African Republic | √ | √ |  |  |  |
| AF192902/1973 | India | √ | √ |  |  |  |
| AF192901/1963 | India | √ | √ |  |  |  |
| AF192900/1996 | Thailand | √ | √ |  |  |  |
| AF192899/1978 | Thailand | √ | √ |  |  |  |
| AF192898/1975 | Thailand | √ | √ |  |  |  |
| AF192897/1995 | Thailand | √ | √ |  |  |  |
| AF192895/1985 | Philippines | √ | √ |  |  |  |
| AF192896/1988 | Thailand | √ | √ |  |  |  |
| AF192894/1985 | Indonesia | √ | √ |  |  |  |
| AF192892/1983 | Senegal | √ | √ | √ | √ | √ |
| AF192891/1966 | Senegal | √ | √ | √ | √ | √ |
| DQ489787/2006 | Hong Kong | √ | √ |  |  |  |
| EF559252/2007 | India | √ | √ | √ | √ | √ |
| FJ445509/2008 | Singapore | √ | √ |  |  |  |
| FJ445508/2008 | Singapore | √ | √ |  |  |  |
| FJ445500/2008 | Singapore | √ | √ |  |  |  |
| FJ445493/2008 | Singapore | √ | √ | √ | √ | √ |
| FJ445488/2008 | Singapore | √ | √ | √ | √ | √ |
| FJ445487/2008 | Singapore | √ | √ | √ | √ | √ |
| FJ445486/2007 | Singapore | √ | √ | √ | √ | √ |
| FJ445485/2007 | Singapore | √ | √ | √ | √ | √ |
| FJ445483/2008 | Singapore | √ | √ | √ | √ | √ |
| FJ445482/2008 | Singapore | √ | √ |  |  |  |
| FJ445480/2008 | Singapore | √ | √ |  |  |  |
| FJ445475/2008 | Singapore | √ | √ | √ | √ | √ |
| GQ229489/2009 | India | √ | √ | √ | √ | √ |
| GQ229488/2009 | India | √ | √ | √ | √ | √ |
| GQ229486/2009 | India | √ | √ |  |  |  |
| GQ996379/2006 | India | √ | √ |  |  |  |
| GQ996378/2006 | India | √ | √ |  |  |  |
| GQ996377/2006 | India | √ | √ |  |  |  |
| GQ996376/2008 | India | √ | √ |  |  |  |
| GQ996375/2008 | India | √ | √ |  |  |  |
| GQ996374/2008 | India | √ | √ |  |  |  |
| GQ996373/2008 | India | √ | √ |  |  |  |
| GQ996372/2008 | India | √ | √ | √ | √ | √ |
| GQ996371/2008 | India | √ | √ |  |  |  |
| GQ996370/2008 | India | √ | √ |  |  |  |
| GU969242/2010 | East Timor | √ | √ | √ | √ | √ |
| JF264892/2009 | India | √ | √ |  |  |  |
| JF264891/2008 | India | √ | √ |  |  |  |
| JF264890/2007 | India | √ | √ |  |  |  |
| KC969208/2007 | India | √ | √ |  |  |  |
| KC969207/1976 | India | √ | √ | √ | √ | √ |
| AM258990/2006 | Reunion | √ | √ |  |  |  |
| AY549584/2004 | Democratic Republic of the Congo | √ | √ | √ | √ | √ |
| AY726732/1983 | Senegal | √ | √ |  |  |  |
| AB860301/2013 | Philippines | √ | √ | √ | √ | √ |
| AY549583/2004 | Democratic Republic of the Congo | √ | √ | √ | √ | √ |
| AB455493/2006 | Sri Lanka | √ | √ |  |  |  |
| AB455494/2006 | Sri Lanka | √ | √ |  |  |  |
| DQ443544/2006 | Reunion | √ | √ |  |  |  |
| DQ480364/2006 | Mayotte | √ | √ |  |  |  |
| KP164571/2014 | Brazil | √ | √ |  |  |  |
| KP164567/2014 | Brazil | √ | √ |  |  |  |
| KP851709/2014 | Mexico | √ | √ |  |  |  |
| FJ513631/2008 | Sri Lanka | √ | √ |  |  |  |
| FJ513632/2008 | Sri Lanka | √ | √ |  |  |  |
| FJ513635/2008 | Sri Lanka | √ | √ |  |  |  |
| FJ513636/2008 | Sri Lanka | √ | √ | √ | √ | √ |
| FJ513641/2008 | Sri Lanka | √ | √ | √ | √ | √ |
| FJ513642/2008 | Sri Lanka | √ | √ | √ | √ | √ |
| FJ513648/2008 | Sri Lanka | √ | √ |  |  |  |
| FJ513650/2008 | Sri Lanka | √ | √ |  |  |  |
| FJ513651/2008 | Sri Lanka | √ | √ |  |  |  |
| FJ513652/2008 | Sri Lanka | √ | √ |  |  |  |
| FJ513653/2008 | Sri Lanka | √ | √ | √ | √ | √ |
| FJ513654/2008 | Sri Lanka | √ | √ |  |  |  |
| FJ513655/2008 | Sri Lanka | √ | √ |  |  |  |
| FJ513656/2008 | Sri Lanka | √ | √ |  |  |  |
| FJ513657/2008 | Sri Lanka | √ | √ | √ | √ | √ |
| FJ513658/2008 | Sri Lanka | √ | √ |  |  |  |
| FJ513659/2008 | Sri Lanka | √ | √ |  |  |  |
| FJ513660/2008 | Sri Lanka | √ | √ |  |  |  |
| FJ513661/2008 | Sri Lanka | √ | √ |  |  |  |
| FJ513662/2008 | Sri Lanka | √ | √ |  |  |  |
| FJ513663/2008 | Sri Lanka | √ | √ |  |  |  |
| FJ513664/2008 | Sri Lanka | √ | √ |  |  |  |
| FJ513665/2008 | Sri Lanka | √ | √ |  |  |  |
| FJ513666/2008 | Sri Lanka | √ | √ |  |  |  |
| FJ513667/2008 | Sri Lanka | √ | √ |  |  |  |
| FJ513668/2008 | Sri Lanka | √ | √ |  |  |  |
| FJ513669/2008 | Sri Lanka | √ | √ |  |  |  |
| FJ513670/2008 | Sri Lanka | √ | √ |  |  |  |
| FJ513671/2008 | Sri Lanka | √ | √ |  |  |  |
| FJ513672/2008 | Sri Lanka | √ | √ |  |  |  |
| FJ513673/2008 | Sri Lanka | √ | √ |  |  |  |
| FJ513674/2008 | Sri Lanka | √ | √ |  |  |  |
| FJ513675/2008 | Sri Lanka | √ | √ | √ | √ | √ |
| FJ513679/2008 | Sri Lanka | √ | √ |  |  |  |
| FJ807887/2007 | Indonesia | √ | √ |  |  |  |
| FJ807897/2007 | Indonesia | √ | √ |  |  |  |
| FJ807898/2008 | Bangladesh | √ | √ | √ | √ | √ |
| FJ445426/2008 | Sri Lanka | √ | √ | √ | √ | √ |
| GQ428210/2006 | India | √ | √ | √ | √ | √ |
| GQ428214/2008 | India | √ | √ | √ | √ | √ |
| GQ428215/2008 | India | √ | √ | √ | √ | √ |
| GU199352/2008 | China | √ | √ | √ | √ | √ |
| GU908223/2009 | Thailand | √ | √ | √ | √ | √ |
| HE806461/2011 | New Caledonia | √ | √ | √ | √ | √ |
| HM045789/1988 | Thailand | √ | √ | √ | √ | √ |
| HM045796/1995 | Thailand | √ | √ | √ | √ | √ |
| HM045814/1975 | Thailand | √ | √ |  |  |  |
| HM045823/1962 | Angola | √ | √ | √ | √ | √ |
| HM045784/1984 | Central African Republic | √ | √ | √ | √ | √ |
| HM045812/1982 | Uganda | √ | √ | √ | √ | √ |
| HM045822/1978 | Central African Republic | √ | √ | √ | √ | √ |
| HQ456251/2005 | Comoros | √ | √ |  |  |  |
| HM045786/1964 | Nigeria | √ | √ | √ | √ | √ |
| HM045818/1981 | Cote d'Ivoire | √ | √ | √ | √ | √ |
| JF274082/2006 | India | √ | √ |  |  |  |
| JX911334/2013 | Netherlands | √ | √ |  |  |  |
| JX911335 /2013 | Netherlands | √ | √ |  |  |  |
| JX911336 /2013 | Netherlands | √ | √ |  |  |  |
| KJ451622/2013 | Micronesia | √ | √ |  |  |  |
| KJ451623/2013 | Micronesia | √ | √ |  |  |  |
| KJ451624/2014 | British Virgin Islands | √ | √ |  |  |  |
| KC488650/2012 | China | √ | √ |  |  |  |
| KF318729/2012 | China | √ | √ |  |  |  |
| KJ679578/2011 | India | √ | √ |  |  |  |
| KC149888/2010 | Netherlands | √ | √ |  |  |  |
| KC149887/2010 | Netherlands | √ | √ |  |  |  |
| KC149889/2010 | Netherlands | √ | √ |  |  |  |
| KC862329/2010 | Indonesia | √ | √ |  |  |  |
| KR559483/2014 | Puerto Rico | √ | √ | √ | √ | √ |
| EU703759/2006 | Malaysia | √ | √ | √ | √ | √ |
| EU703760/2006 | Malaysia | √ | √ | √ | √ | √ |
| KP164568/2014 | Brazil | √ | √ |  |  |  |
| KP164569/2014 | Brazil | √ | √ | √ | √ | √ |
| FJ445510/2008 | Singapore | √ | √ |  |  |  |
| JX088705/2010 | China | √ | √ |  |  |  |
| KJ941050/2006 | USA | √ | √ |  |  |  |
| EU244823/2007 | Italy | √ | √ | √ | √ | √ |
| JQ065886/2010 | China | √ | √ |  |  |  |
| HQ846358/2010 | China | √ | √ |  |  |  |
| KF590567/2010 | Myanmar | √ | √ |  |  |  |
| KF590566/2010 | Myanmar | √ | √ |  |  |  |
| KF590565/2010 | Myanmar | √ | √ | √ | √ | √ |
| KF590564/2010 | Myanmar | √ | √ |  |  |  |
| FJ445484/2008 | Singapore | √ | √ |  |  |  |
| GQ905863/2009 | Thailand | √ | √ |  |  |  |
| EU372006 /2007 | India | √ | √ |  |  |  |
| AM258993/2006 | Reunion | √ | √ |  |  |  |
| HM159388/2010 | India | √ | √ |  |  |  |
| EF027139/2000 | India | √ | √ |  |  |  |
| HM045805/1976 | South Africa | √ | √ | √ | √ | √ |
| AF369024/1953 | Tanzania | √ | √ |  |  |  |
| EU703762 /2006 | Malaysia | √ | √ | √ | √ | √ |
| HM045817/2005 | Senegal | √ | √ |  |  |  |
| KR559481/2014 | Guatemala | √ | √ |  |  |  |
| KP164572/2014 | Brazil | √ | √ |  |  |  |
| KP851710/2014 | Mexico | √ | √ |  |  |  |
| EF027141/1973 | India | √ | √ |  |  |  |
| EF027134/2006 | India | √ | √ |  |  |  |
| EF027135/2006 | India | √ | √ | √ | √ | √ |
| EF027136/2006 | India | √ | √ | √ | √ | √ |
| EF027137/2006 | India | √ | √ |  |  |  |
| EF027138/2006 | India | √ | √ | √ | √ | √ |
| EF051584/2006 | Cameroon | √ | √ | √ | √ | √ |
| EF210157/2006 | India | √ | √ |  |  |  |
| EF451147/2006 | India | √ | √ |  |  |  |
| EF613344/2007 | Gabon | √ | √ | √ | √ | √ |
| EF012359/2006 | Mauritius | √ | √ |  |  |  |
| EF027140/1963 | India | √ | √ | √ | √ | √ |
| EF452493/2007 | Thailand | √ | √ | √ | √ | √ |
| EU441883/2008 | Singapore | √ | √ |  |  |  |
| EU564334/2006 | Mauritius | √ | √ |  |  |  |
| EU564335/2006 | India | √ | √ |  |  |  |
| EU703761/2006 | Malaysia | √ | √ | √ | √ | √ |
| EU037962/2006 | Mauritius | √ | √ |  |  |  |
| FJ445502/2008 | Singapore | √ | √ |  |  |  |
| FJ000068/2006 | India | √ | √ | √ | √ | √ |
| FJ000067/2006 | India | √ | √ |  |  |  |
| FJ000064/2006 | India | √ | √ | √ | √ | √ |
| FJ000063/2006 | India | √ | √ | √ | √ | √ |
| FJ432665/2008 | India | √ | √ |  |  |  |
| FJ445424/2008 | Sri Lanka | √ | √ |  |  |  |
| KF305688/2011 | India | √ | √ |  |  |  |
| KF305690/2011 | India | √ | √ |  |  |  |
| KF305692/2011 | India | √ | √ |  |  |  |
| KF305695/2011 | India | √ | √ |  |  |  |
| KF872195/2013 | Moscow | √ | √ | √ | √ | √ |
| EU224270/2007 | USA | √ | √ | √ | √ | √ |
| KT336778/2010 | India | √ | √ | √ | √ | √ |
| KJ796844/2009 | Russia | √ | √ | √ | √ | √ |
| KJ796846/2009 | India | √ | √ | √ | √ | √ |
| KJ796850/2009 | Thailand | √ | √ | √ | √ | √ |
| KR559485/2014 | Virgin Islands | √ | √ | √ | √ | √ |
| JQ067624/2010 | China | √ | √ | √ | √ | √ |
| JQ861258/2011 | Cambodia | √ | √ | √ | √ | √ |
| JQ861260/2011 | Cambodia | √ | √ | √ | √ | √ |
| JN558834/2008 | India | √ | √ | √ | √ | √ |
| JN558836/2009 | India | √ | √ | √ | √ | √ |
| JQ065890/2010 | China | √ | √ | √ | √ | √ |
| KP003812/2007 | Gabon | √ | √ | √ | √ | √ |
| KP003813/2013 | Congo | √ | √ | √ | √ | √ |
| KR264950/2014 | Puerto Rico | √ | √ | √ | √ | √ |
| FJ445431/2008 | Singapore | √ | √ | √ | √ | √ |
| KM985982/2014 | Tonga | √ | √ | √ | √ | √ |
| FN295484/2006 | Malaysia | √ | √ | √ | √ | √ |
| HQ846357/2010 | China | √ | √ | √ | √ | √ |
| DQ462749/2006 | Reunion | √ | √ | √ | √ | √ |
| DQ462750/2006 | Reunion | √ | √ | √ | √ | √ |
| AY549575/2004 | Congo | √ | √ | √ | √ | √ |
| AY549582/2004 | Congo | √ | √ | √ | √ | √ |
| AY424803/2000 | India | √ | √ | √ | √ | √ |
| HM045800/1985 | Philippines | √ | √ | √ | √ | √ |
| L37661/NA | USA | √ | √ | √ | √ | √ |
| KM673291/2013 | Indonesia | √ | √ | √ | √ | √ |
| HM045787/1966 | Senegal | √ | √ | √ | √ | √ |
| HM045802/1995 | Thailand | √ | √ | √ | √ | √ |
| KP685237/2013 | Singapore | √ | √ | √ | √ | √ |
| HM045805/1976 | South Africa | √ | √ | √ | √ | √ |
| HM045806/1986 | India | √ | √ | √ | √ | √ |
| KP164570/2014 | Brazil | √ | √ | √ | √ | √ |
| HM045793 | Central African Republic | √ | √ | √ | √ | √ |
| HM045798/1966 | Senegal | √ | √ | √ | √ | √ |
| HM045816/1966 | Senegal | √ | √ | √ | √ | √ |
| JQ943714/2009 | Senegal | √ | √ | √ | √ | √ |
| HM045815/1979 | Senegal | √ | √ | √ | √ | √ |
| GQ168719/2008 | Australia | √ | √ | √ | √ | √ |
| KF818473/2011 | India | √ | √ | √ | √ | √ |
| JQ943718/2009 | Senegal | √ | √ | √ | √ | √ |
| JX442959/2006 | India | √ | √ | √ | √ | √ |
| AB857743/2010 | Thailand | √ | √ | √ | √ | √ |
| AB857777/2010 | Thailand | √ | √ | √ | √ | √ |
| FJ705371/2008 | India | √ | √ | √ | √ | √ |
| EF451143/2006 | India | √ | √ | √ | √ | √ |
| EF451149/2006 | India | √ | √ | √ | √ | √ |
| EF451145/2006 | India | √ | √ | √ | √ | √ |
| FR846304/2009 | France | √ | √ | √ | √ | √ |
| KT336783/2012 | India | √ | √ | √ | √ | √ |
| FJ705370/2008 | India | √ | √ | √ | √ | √ |
| FR846305/2009 | France | √ | √ | √ | √ | √ |
| FJ705369/2008 | India | √ | √ | √ | √ | √ |
| AB857781/2010 | Thailand | √ | √ | √ | √ | √ |
| KC879559/2013 | Indonesia | √ | √ | √ | √ | √ |
| KC879565/2013 | Indonesia | √ | √ | √ | √ | √ |
| KC879568/2013 | Indonesia | √ | √ | √ | √ | √ |
| KC879569/2013 | Indonesia | √ | √ | √ | √ | √ |
| KC879571/2013 | Indonesia | √ | √ | √ | √ | √ |
| KC879577/2013 | Indonesia | √ | √ | √ | √ | √ |
| KC879578/2013 | Indonesia | √ | √ | √ | √ | √ |
| KC731586/2012 | Bhutan | √ | √ | √ | √ | √ |
| KC524772/2012 | Papua New Guinea | √ | √ | √ | √ | √ |
| KC810970/2009 | South Korea | √ | √ | √ | √ | √ |
| HQ848080/2008 | India | √ | √ | √ | √ | √ |
| EU192143/2007 | Indonesia | √ | √ | √ | √ | √ |
| HQ848081/2009 | India | √ | √ | √ | √ | √ |
| KT336794/2013 | India | √ | √ | √ | √ | √ |
| KT336787/2012 | India | √ | √ | √ | √ | √ |
| KT336788/2012 | India | √ | √ | √ | √ | √ |
| JQ065889/2010 | China | √ | √ | √ | √ | √ |
| KT211025/2014 | Columbia | √ | √ | √ | √ | √ |
| KT211028/2014 | Columbia | √ | √ | √ | √ | √ |
| GQ143741/2008 | Singapore | √ | √ | √ | √ | √ |
| KT211038/2014 | Columbia | √ | √ | √ | √ | √ |
| KP702204/2012 | Vietnam | √ | √ | √ | √ | √ |
| JN038571/2010 | India | √ | √ | √ | √ | √ |
| KP702205/2012 | Vietnam | √ | √ | √ | √ | √ |
| GQ143746/2008 | Singapore | √ | √ | √ | √ | √ |
| KC731581/2012 | Bhutan | √ | √ | √ | √ | √ |
| JN661152/2009 | Thailand | √ | √ | √ | √ | √ |
| HM045809/2006 | Congo | √ | √ | √ | √ | √ |
| HM045788/1973 | India | √ | √ | √ | √ | √ |
| HM045808/1978 | Thailand | √ | √ | √ | √ | √ |
| HM045797/1985 | Indonesia | √ | √ | √ | √ | √ |
| GU189061/2006 | Sri Lanka | √ | √ | √ | √ | √ |
| FJ807886/2007 | Indonesia | √ | √ | √ | √ | √ |
| FJ513677/2008 | Sri Lanka | √ | √ | √ | √ | √ |
| GQ229487/2008 | India | √ | √ | √ | √ | √ |
| MF573005/2015 | India | √ | √ |  |  |  |
| MF573008/2015 | India | √ | √ |  |  |  |
| MH400249/2017 | China | √ | √ |  |  |  |
| KY704002/2015 | Managua | √ | √ |  |  |  |
| KY680382/2014 | USA | √ | √ |  |  |  |
| KY680366/2015 | USA | √ | √ |  |  |  |
| MF773564/2016 | Philippines | √ | √ |  |  |  |
| MF740880/2017 | Pakistan | √ | √ |  |  |  |
| LC146714/2016 | Cuba | √ | √ |  |  |  |
| KX496989/2016 | Colombia | √ | √ |  |  |  |
| KX228391/2016 | Brazil | √ | √ |  |  |  |
| MF503628/2016 | Hong Kong | √ | √ |  |  |  |
| KY124329/2016 | Brazil | √ | √ |  |  |  |
| MH823668/2017 | Brazil | √ | √ |  |  |  |

**Supplementary table S2: Molecular clocks and their corresponding tree priors with log marginal values generated through BEAST.**

| Molecular Clock | Tree Prior | Log marginal likelihood (stepping sampling) | Log marginal likelihood (path sampling) |
| --- | --- | --- | --- |
| Strict | Constant | -4909.5 | -4908.1 |
|  | lognormal | -4900.8 | -4899.6 |
|  | **Skyline** | **-4901.2** | **-4887.7** |
| Relaxed | Constant | -6601.3 | -6600.0 |
|  | lognormal | -6606.9 | -6604.7 |
|  | Skyline | -6595.5 | -6594.1 |

**Supplementary table S3: Scores of various methods for B-cell epitope prediction**

| **S.No** | **Prediction method** | **Minimum score** | **Maximum**  **score** | **Average score** | **Threshold value** |
| --- | --- | --- | --- | --- | --- |
| 1 | Bepipred linear epitope | -3.2 | 1.8 | 0.089 | 0.09 |
| 2 | Chou and Fasman β-turn | 0.5 | 0.09 | 0.9 | 1.0 |
| 3 | Emini surface accessibility | 1.0 | 6.0 | 0.03 | 1.4 |
| 4 | Karplus and Schulz | 0.8 | 1.095 | 0.9 | 1.5 |
| 5 | Kolaskar and Tongaonkar | 0.9 | 1.3 | 1.2 | 1.6 |
| 6 | Parker Hydrophilicity | -7.2 | 5.9 | 1.46 | 1.48 |

**Supplementary table S4*:* Potential MHC-I epitopes of the E1 protein of Chikungunya virus**

| **Epitope** | **Position** | **Alleles** | **Ic50<200, combined score** | **NetCTL score of HLA supertypes** | **Immunogenicity**  **Score** | **Epitope Conservancy (Max. identity %)** |
| --- | --- | --- | --- | --- | --- | --- |
| YPFMWGGAY | 85-93 | HLA-B*35:01, | 2.12, 1.92 | A3:1.07 B7:1.96 B62:0.9 | 0.16036 | 100 |
|  |  | HLA-B*15:02, | 1.21, 15.59 |  |  |  |
|  |  | HLA-B*18:01, | 0.30, 126.37 |  |  |  |
|  |  | HLA-B*53:01, | 0.92, 30.62 |  |  |  |
|  |  | HLA-B*56:01, | 0.16, 177.37 |  |  |  |
|  |  | HLA-B*35:03 | 0.15, 182.10 |  |  |  |
| KVFTGVYPF | 79-87 | HLA-B*15:25, | 1.71, 4.64 | A3:1.1 A26:1.55 B58:0.9 B62:0.9 | 0.13932 | 100 |
|  |  | HLA-B*15:01, | 1.05, 21.20 |  |  |  |
|  |  | HLA-B*15:02, | 0.65, 54.02 |  |  |  |
|  |  | HLA-B*35:01, | 0.45, 84.32 |  |  |  |
|  |  | HLA-A*32:01, | 1.96, 2.63 |  |  |  |
|  |  | HLA-B*58:01 | 0.66, 52.28 |  |  |  |
| FMWGGAYCF | 87-95 | HLA-A*29:02 | 1.47, 20.06 | A2:1.5 A26:1.03 B62: 1.3 | 0.14121 | 100 |
|  |  | HLA-A*23:01 | 1.10, 46.46 |  |  |  |
|  |  | HLA-A*02:06 | 1.45, 20.56 |  |  |  |
|  |  | HLA-A*02:01 | 1.34, 26.88 |  |  |  |
|  |  | HLA-B*15:25 | 1.52, 17.85 |  |  |  |
|  |  | HLA-B*15:02 | 0.87, 78.80 |  |  |  |
|  |  | HLA-B*15:01 | 0.84, 83.71 |  |  |  |
|  |  | HLA-B*35:01 | 1.09, 48.10 |  |  |  |

**Supplementary table S5: Potential MHC-II epitopes of the E1 protein of Chikungunya virus**

| **Peptide** | **Interacting alleles with IC50<50 (and their respective PREDIVAC score)** | **Amino acid position** |
| --- | --- | --- |
| VHSMTNAVT | HLA-DRB1*01:02 (90.9), HLA-DRB1*01:01 (84.1), HLA-DRB1*11:04 (84.53) | 330-338 |
| *WLKERGASL* | HLA-DRB1*01:01 (88.6), HLA-DRB1*01:02 (84.3) | 243-251 |
| IKYAVSKKG | HLA-DRB1*08:04 (85.6), HLA-DRB1*08:06 (81.35) | 318-326 |
| YKTLVNRPG | HLA-DRB1*01:01 (88.0), HLA-DRB1*13:21 (87.1), HLA-DRB1*01:02 (86.9), HLA-DRB1*11:04 (87.24), HLA-DRB1*11:28 (88.7), HLA-DRB1*13:05 (88.7), HLA-DRB1*11:01 (92) | 15-23 |
